# Multiple decades of stocking has resulted in limited hatchery introgression in wild brook trout (*Salvelinus fontinalis*) populations of Nova Scotia

**DOI:** 10.1111/eva.12923

**Published:** 2020-02-20

**Authors:** Sarah J. Lehnert, Shauna M. Baillie, John MacMillan, Ian G. Paterson, Colin F. Buhariwalla, Ian R. Bradbury, Paul Bentzen

**Affiliations:** ^1^ Fisheries and Oceans Canada Northwest Atlantic Fisheries Centre St. John's NL Canada; ^2^ Marine Gene Probe Lab Biology Department Dalhousie University Halifax NS Canada; ^3^ Inland Fisheries Division Nova Scotia Department of Fisheries and Aquaculture Pictou NS Canada

**Keywords:** brook trout, domestication, hatchery stocking, hybridization, introgression, microsatellites

## Abstract

Many populations of freshwater fishes are threatened with losses, and increasingly, the release of hatchery individuals is one strategy being implemented to support wild populations. However, stocking of hatchery individuals may pose long‐term threats to wild populations, particularly if genetic interactions occur between wild and hatchery individuals. One highly prized sport fish that has been heavily stocked throughout its range is the brook trout (*Salvelinus fontinalis*). In Nova Scotia, Canada, hatchery brook trout have been stocked since the early 1900s, and despite continued stocking efforts, populations have suffered declines in recent decades. Before this study, the genetic structure of brook trout populations in the province was unknown; however, given the potential negative consequences associated with hatchery stocking, it is possible that hatchery programs have adversely affected the genetic integrity of wild populations. To assess the influence of hatchery supplementation on wild populations, we genotyped wild brook trout from 12 river systems and hatchery brook trout from two major hatcheries using 100 microsatellite loci. Genetic analyses of wild trout revealed extensive population genetic structure among and within river systems and significant isolation‐by‐distance. Hatchery stocks were genetically distinct from wild populations, and most populations showed limited to no evidence of hatchery introgression (<5% hatchery ancestry). Only a single location had a substantial number of hatchery‐derived trout and was located in the only river where a local strain is used for supplementation. The amount of hatchery stocking within a watershed did not influence the level of hatchery introgression. Neutral genetic structure of wild populations was influenced by geography with some influence of climate and stocking indices. Overall, our study suggests that long‐term stocking has not significantly affected the genetic integrity of wild trout populations, highlighting the variable outcomes of stocking and the need to evaluate the consequences on a case‐by‐case basis.

## 1 INTRODUCTION

Freshwater fishes are ecologically, culturally, and economically valued in North America, yet these species are threatened by higher extinction rates relative to many other vertebrates (Burkhead, 2012). Many populations have been adversely affected by recent environmental and anthropogenic changes (Lynch et al., 2016), and one management strategy to conserve these populations is to supplement them with captive‐bred individuals. The goal of these supplementation programs can be to increase abundance to aid in population recovery or alternatively these programs can be implemented to reduce pressure on wild populations by increasing the number of individuals available for harvest (Naish et al., 2007).

While supplementation can immediately increase the total abundance of fish in a system, there is much controversy regarding the overall effect on wild populations (Araki & Schmid, 2010; Brannon et al., 2004; Naish et al., 2007). Salmonid species represent a large proportion of artificially propagated fishes (Araki, Berejikian, Ford, & Blouin, 2008; Hilborn, 2007; Lackey, Lach, & Duncan, 2006), yet increasing evidence finds that hatchery salmonids are often less fit than wild conspecifics (Araki & Schmid, 2010). The hatchery environment can cause phenotypic and genetic changes; for example, captive rearing has been documented to lead to a reduction in reproductive success (Araki, Cooper, & Blouin, 2007), a decrease in egg size (Heath, Heath, Bryden, Johnson, & Fox, 2003), changes in behavior (Brown & Laland, 2001; Fleming & Gross, 1993; Sundström, Petersson, Höjesjö, Johnsson, & Järvi, 2004), lower genetic diversity (Christie, Marine, French, Waples, & Blouin, 2012), heritable changes in gene expression (Christie, Marine, Fox, French, & Blouin, 2016), and epigenetic modification (i.e., DNA methylation) (Le Luyer et al., 2017). Depending on the program objectives, captive rearing can result in intentional and unintentional changes that may produce fish that are maladapted under natural conditions; thus, the release of captive‐reared individuals can have negative consequences if these individuals interact and hybridize with wild individuals (Amoroso, Tillotson, & Hilborn, 2017; Araki, Cooper, et al., 2007; Glover et al., 2017; McGinnity et al., 2003). This can be especially problematic when hatchery strains are derived from nonlocal populations, thus further reducing the likelihood that these individuals will be suited to local environments (Laikre, Schwartz, Waples, & Ryman, 2010; Weigel, Adams, Jepson, Waits, & Caudill, 2019).

The release of hatchery salmonids can pose both ecological and genetic risks to wild populations, where ecological impacts can occur through interactions related to competition, predation, and disease transfer (Krueger & May, 1991; Naish et al., 2007; Rand et al., 2012). Genetic impacts on wild populations can occur through hybridization and introgression whereby such events can reduce genetic diversity (Christie et al., 2012; Gossieaux, Bernatchez, Sirois, & Garant, 2019), introduce maladaptive alleles (Ferchaud, Laporte, Perrier, & Bernatchez, 2018), break down coadapted gene complexes (Waples, 1991), alter gene expression patterns (Lamaze, Garant, & Bernatchez, 2013), and genetically homogenize natural populations (Marie, Bernatchez, & Garant, 2010). These genetic interactions between hatchery and wild salmonids may pose long‐term threats to wild populations (Muhlfeld et al., 2017; Naish et al., 2007; Willoughby & Christie, 2019) as the genetic legacy of stocking events may persist in populations long after stocking is terminated (Jones, Clay, & Danzmann, 1996; Muhlfeld et al., 2017; Willoughby, Harder, Tennessen, Scribner, & Christie, 2018).

Brook trout (*Salvelinus fontinalis*) is a highly prized sport fish that has been extensively stocked throughout its native range in eastern North America as well as other regions throughout the world (Kerr, 2000; Kitano, 2004; Lecomte, Beall, Chat, Davaine, & Gaudin, 2013; MacCrimmon & Campbell, 1969). While stocking of brook trout has occurred for more than a century in some regions (Kerr, 2000; MacCrimmon & Campbell, 1969), many populations have declined in recent decades (Hudy, Thieling, Gillespie, & Smith, 2008; MacMillan, Caissie, Marshall, & Hinks, 2008; Nova Scotia Department of Agriculture & Fisheries, 2005; Stranko et al., 2008). Brook trout populations are threatened by habitat alteration, warming climate, overexploitation, and the introduction of invasive species (Hudy et al., 2008; MacCrimmon & Campbell, 1969; MacMillan et al., 2008; Stranko et al., 2008), and stocking can be a promising management tool to conserve populations and/or increase recreational opportunities. However, the impacts of stocking on wild brook trout populations can be variable. In some systems, hatchery stocking has led to extensive hybridization between hatchery and wild brook trout (Harbicht, Alshamlih, Wilson, & Fraser, 2014; Lamaze, Sauvage, Marie, Garant, & Bernatchez, 2012) and disrupted the genetic integrity of wild populations (Gossieaux et al., 2019; Lamaze et al., 2013, 2012; Marie et al., 2010). In these cases, the probability of hatchery introgression can be influenced by various factors, including the timing and intensity of stocking events, number of years since stocking, the size of the watershed, and environmental variables such as dissolved oxygen, temperature, and pH (Harbicht et al., 2014; Létourneau et al., 2018; Marie, Bernatchez, & Garant, 2012). These studies represent systems where stocking events have occurred in lakes; however, other studies conducted on river systems have often found limited genetic impact of hatchery stocking on brook trout populations (Jones et al., 1996; Kazyak, Rash, Lubinski, & King, 2018; Kelson, Kapuscinski, Timmins, & Ardren, 2015; White, Miller, Dowell, Bartron, & Wagner, 2018). For example, White et al. (2018) found that less than 6% of wild‐caught brook trout showed evidence of hatchery ancestry despite recurrent stocking efforts.

In Nova Scotia, brook trout is one of the most sought after sport fishes, and to meet the needs of recreational anglers, brook trout have been stocked since at least 1904 (Department of Marine & Fisheries, 1905) with detailed records extending back to 1974 (Alexander, 1977; Nova Scotia Government, 2018). Despite stocking efforts, recreational catches have declined by approximately 60% in recent decades, corresponding to reduced angling effort as well as impacts associated with environmental and anthropogenic changes (Heggelin, 2008; Nova Scotia Department of Agriculture & Fisheries, 2005). To date, the population genetic structure of brook trout in the province of Nova Scotia had not been reported; however, brook trout populations are often found to be highly genetically structure within and among river systems (Ferchaud et al., 2019; Ward, Woodwark, & Skibinski, 1994), and thus, high levels of genetic structure may be expected in Nova Scotia rivers. Nonetheless, given the extensive stocking that has occurred, it is possible that these programs have affected the genetic integrity of populations (Marie et al., 2010), which could influence their vulnerability. To assess the genetic effect of hatchery supplementation on wild brook trout populations in Nova Scotia, we genotyped hatchery and wild brook trout from 12 river systems that vary in stocking intensity. The main objectives of our study are threefold. First, we aim to characterize genetic diversity and structure of wild brook trout populations that have experienced varying levels of supplementation. Using this information, our second objective is to quantify genetic differentiation between the hatchery and wild populations and determine the level of hatchery introgression in the wild. Finally, our third objective is to determine which environmental and anthropogenic variables influence genetic structure and the level of hatchery introgression in wild populations. Our study will provide information on connectivity and diversity of wild trout populations and determine to what extent populations have been genetically affected by long‐term hatchery stocking programs. As animal populations worldwide continue to be threatened with losses (Burkhead, 2012; Ceballos, Ehrlich, & Dirzo, 2017), supplementation of captive‐bred individuals remains an important approach for supporting and conserving these populations (Ebenhard, 1995; Naish et al., 2007; Seddon, Armstrong, & Maloney, 2007). Therefore, it is necessary that we understand the genetic consequences of releasing captive‐bred individuals and identify the factors that may increase the probability of introgression in the wild to help predict the outcomes of supplementation.

## 2 MATERIALS AND METHODS

### 2.1 Fish sampling and tissue collection

**Figure 1:**
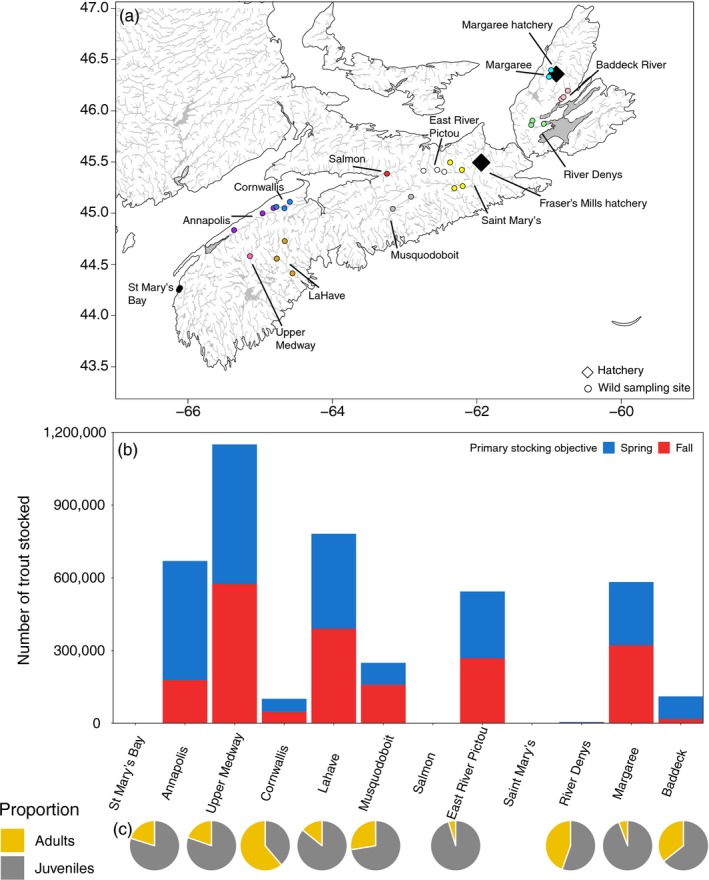
(a) Map of brook trout (*Salvelinus fontinalis*) sampling sites in Nova Scotia, Canada. Points are colored by river system. Black diamonds indicate the two hatchery locations that were also sampled in the study. (b) Total number of brook trout stocked in each river system calculated from provincial records dating back to 1976. Colors indicate the number of trout stocked in spring (blue) and fall (red) programs. See Figure S1 for size distributions of trout stocking. (c) Pie charts showing the proportion of adults (yellow) and juveniles (gray) stocked overall within each system

Wild brook trout were sampled by angling and backpack electrofishing from multiple sites within 12 river systems across the province of Nova Scotia, Canada, between 2016 and 2018 (Table S1 and Figure 1a). Sampled trout were ages 1+ and 2+ to reduce the probability of collecting siblings, which is likely to be higher during early life stages provided less opportunity for selection to operate. Nonetheless, we did not measure relatedness among individuals and therefore did not attempt to remove siblings because this can result in a loss of information in population genetic datasets (Waples & Anderson, 2017). Young‐of‐the‐year (YOY) were also captured at one site (Varner Brook), and these samples were kept separate from older fish (Table S1). A distance of >1 km separated the majority of sites within a system, which is greater than the home range of brook trout reported in earlier studies (Gowan & Fausch, 1996; Hartman & Logan, 2010; Moore, Larson, & Ridley, 1985). In addition, samples from each site were collected within a single year and not over multiple years. Adipose fin clips were taken and air‐dried in paper envelopes for later DNA analyses. Hatchery brook trout are stocked within many of these river systems (Figure 1b,c and Figure S1; see below), and at one sampling site (Lake O'Law Brook, Margaree River), it was suspected that some wild‐caught fish might be hatchery origin due to evidence of fin erosion. At this site, samples with fin erosion were separated from those without fin erosion for analyses (Table S1).

Hatchery produced brook trout are stocked in many Nova Scotia water bodies in the fall and spring (see Figure 1b and Table 2). The spring stocking program includes the use of fry to supplement recruitment of trout to the fishery and sizeable trout for immediate harvest. The fall stocking program stocks sizeable trout to support winter angling and fingerlings that can grow to be harvested later (Nova Scotia Department of Agriculture & Fisheries, 2005) (see Figure S1 for size distributions and Figure 1c for proportion of adults (≥1 year posthatch) and juveniles stocked). Fin clips were collected from hatchery brook trout at Fraser's Mills and Margaree Fish hatchery facilities, two of the three provincial hatcheries (see Table 1 and Figure 1a). We did not collect samples from the third hatchery operated by the province, McGowan Lake hatchery, which stocks trout in the western part of Nova Scotia. This hatchery was established in 1987 with trout originating from Fraser's Mills hatchery and continues to receive eggs from Fraser's Mills hatchery each year; thus, we expect the two hatcheries to be genetically similar. The Fraser's Mills hatchery was established in 1928 with trout from different sources including Saint John River hatchery, New Brunswick, Canada, and the American Fish Culture Company, Rhode Island, USA (Department of Marine & Fisheries, 1929). To date, no studies have investigated the genetic origins of this hatchery stock. Samples were collected and preserved from four strains maintained at the Fraser's Mills hatchery, which include Flat Lake strain, Timber Lake strain, Fraser's Mills domestic strain, and Sea Trout strain. Additional hatchery samples were collected from a trout derby in Pictou, NS, where strains from Fraser's Mills were stocked in a pond. At Margaree Fish hatchery, samples were collected and preserved from the single strain maintained at the hatchery. The Margaree Fish hatchery was established in 1902 and currently uses locally sourced wild brook trout from the Margaree River for hatchery production (D. Murrant, Manager of Fisheries Enhancement, Nova Scotia Department of Fisheries and Aquaculture; pers. comm.).

**Table 1:** Genetic diversity estimates for wild brook trout (*Salvelinus fontinalis*) populations in Nova Scotia, Canada, based on 100 microsatellites

Code	River system	*N*	*F*_IS_			*H*_o_			*H*_e_			*N*_e_		
			Mean	CI_L_	CI_U_	Mean	CI_L_	CI_U_	Mean	CI_L_	CI_U_	*N_e_*	CI_L_	CI_U_
Duf	St Mary's Bay	50	0.020	−0.018	0.058	0.33	0.28	0.38	0.34	0.29	0.38	50.4	43.2	59.5
Bou1	St Mary's Bay	29	0.003	−0.042	0.049	0.39	0.34	0.43	0.39	0.35	0.44	60.4	48.2	79.6
Bou2	St Mary's Bay	20	0.004	−0.043	0.051	0.36	0.31	0.41	0.36	0.32	0.41	40.9	32.2	55.1
FO	Annapolis River	44	0.005	−0.026	0.036	0.33	0.28	0.38	0.33	0.28	0.38	328.9	206.7	760.6
WA	Annapolis River	46	0.004	−0.029	0.037	0.35	0.31	0.40	0.36	0.31	0.41	313.8	209	607.2
Par	Annapolis River	47	−0.006	−0.034	0.022	0.35	0.30	0.40	0.35	0.30	0.40	168.3	132.4	228
UM	Upper Medway	128	0.055	0.035	0.074	0.31	0.27	0.36	0.33	0.29	0.38	263.2	220.6	323.2
RA	Cornwallis River	46	−0.006	−0.035	0.023	0.35	0.30	0.40	0.35	0.29	0.40	174.8	134.1	247
Roc	Cornwallis River	52	0.012	−0.018	0.042	0.36	0.31	0.41	0.36	0.31	0.41	57.8	51.5	65.4
BR	Cornwallis River	44	0.003	−0.030	0.036	0.34	0.29	0.38	0.33	0.29	0.38	549.8	277	9,930.6
Var	LaHave	38	0.013	−0.021	0.047	0.32	0.28	0.37	0.33	0.28	0.37	107.5	84.2	146
VarYOY	LaHave	12	−0.067	−0.135	0.001	0.36	0.30	0.41	0.33	0.28	0.37	26.8	20	39
BU	LaHave	68	0.004	−0.025	0.033	0.35	0.31	0.40	0.36	0.31	0.40	66.1	59.5	74
CO	LaHave	48	0.053	0.021	0.086	0.35	0.30	0.40	0.37	0.32	0.41	72	63.7	82.3
Far	Salmon River Truro	48	0.005	−0.031	0.042	0.34	0.29	0.39	0.34	0.29	0.39	208.4	141.7	376.5
MC	Musquodoboit River	47	0.015	−0.014	0.044	0.34	0.29	0.39	0.35	0.30	0.39	268.1	177.7	521.6
GE	Musquodoboit River	46	−0.015	−0.054	0.023	0.33	0.28	0.38	0.34	0.29	0.39	89.8	74	112.7
BI	East River Pictou	47	0.013	−0.022	0.047	0.39	0.34	0.44	0.39	0.34	0.44	373.2	242.3	779.9
GL	East River Pictou	47	0.026	−0.015	0.067	0.30	0.26	0.34	0.30	0.26	0.35	10.1	9.2	11.1
Tho	East River Pictou	46	−0.010	−0.036	0.017	0.40	0.35	0.45	0.39	0.35	0.44	782	372	∞
MO	Saint Mary's River (East)	42	−0.010	−0.038	0.019	0.35	0.30	0.39	0.34	0.29	0.38	118.5	93.6	158.8
Kel	Saint Mary's River	24	−0.067	−0.108	−0.025	0.32	0.26	0.37	0.29	0.25	0.34	137.9	79.7	438.2
GR	Saint Mary's River (East)	47	0.003	−0.037	0.043	0.22	0.18	0.26	0.22	0.18	0.26	88.5	65.6	130.5
Cla	Saint Mary's River	20	−0.038	−0.074	−0.003	0.37	0.31	0.42	0.35	0.30	0.40	−774.1	402.3	∞
SA	River Denys	55	−0.001	−0.022	0.020	0.39	0.34	0.43	0.39	0.34	0.43	−1,729.3	2,061.6	∞
AL	River Denys	45	0.004	−0.029	0.037	0.39	0.34	0.44	0.39	0.35	0.44	558.1	318.8	2,044
RD	River Denys	50	0.027	−0.007	0.061	0.38	0.33	0.43	0.39	0.35	0.44	1,834.6	533.5	∞
LakH	Margaree River	31	0.001	−0.033	0.036	0.38	0.34	0.43	0.39	0.34	0.43	109.3	83.8	154.5
LakW	Margaree River	55	0.010	−0.023	0.043	0.39	0.35	0.44	0.39	0.35	0.43	1,641	529.3	∞
PO	Margaree River	48	0.010	−0.019	0.039	0.40	0.36	0.45	0.40	0.36	0.45	887.8	388.8	∞
HA	Baddeck River	50	0.006	−0.021	0.034	0.38	0.34	0.43	0.39	0.35	0.44	3,188.5	581.3	∞
Ang	Baddeck River	48	−0.009	−0.035	0.017	0.41	0.36	0.45	0.40	0.36	0.45	709.7	371.4	6,024.4
MI	Baddeck River	52	0.001	−0.025	0.027	0.39	0.34	0.44	0.39	0.34	0.43	341.4	233.6	616.1

Note

Estimates for inbreeding coefficient (*F*_IS_), observed heterozygosity (*H*_o_), expected heterozygosity (*H*_e_), and effective population size (*N*_e_) are provided with 95% confidence intervals (CI). *N*_e_ was calculated using an allele frequency cutoff (*P*‐crit) of 0.01, and the corresponding confidence intervals were adjusted using the jackknife method.

### 2.2 DNA extraction and microsatellite sequencing

DNA was extracted from tissue using a solid‐phase technique modified from Elphinstone, Hinten, Anderson, and Nock (2003). Briefly, tissue was digested with proteinase K, and the DNA was bound to silica fines in a chaotropic salt, purified using 384‐well filtration plates, and eluted in a low‐salt buffer (1× TE). DNA was multiplexed and indexed following Zhan et al. (2017) using 4 separate multiplex reactions of 19, 23, 25, and 33 loci per multiplex. A total of 100 microsatellite loci (Table S2) were selected from previously developed “legacy” loci (*n* = 33) and from newly identified loci (*n* = 67) in the Arctic charr (*Salvelinus alpinus*) genome. The thirty‐three legacy loci included 5 loci from brook trout *S. fontinalis* (King, Lubinski, Burnham‐Curtis, Stott, & Morgan, 2012) and 28 loci from Atlantic salmon *Salmo salar* (see Bradbury et al. (2018) for microsatellite loci and Lien et al. (2016) for Atlantic salmon genome used for primer design). The sixty‐seven newly developed loci were designed from the Arctic charr genome (Christensen et al., 2018) and included 42 loci used in Layton et al. (in press) for Arctic charr and 25 loci that were uninformative in Arctic charr but proved variable in the present study of brook trout.

Pooled libraries were sequenced on an Illumina MiSeq at an average depth of 500 reads per sample per locus using 150 cycle v3 reagent kits. Loci were demultiplexed, and alleles were scored using MEGASAT (Zhan et al., 2017). Individuals were filtered from the dataset if the genotyping rate was lower than 70% (*n* = 37 individuals removed). Loci were also examined for missing data; however, all loci were genotyped in >70% of individuals, and thus, no loci were removed from the analyses. After filtering, a total of 1,520 wild trout and 209 hatchery trout were used for genetic analyses in our study. For wild brook trout sites, all loci had 2 or more alleles with a mean of 7.66 alleles per locus (range 2–33 alleles). In addition, across all wild sites and loci comparisons (*n* = 3,300), only 1% of comparisons showed significant deviations (adjusted alpha = 0.05/33) from Hardy–Weinberg equilibrium (HWE) using the R package *diveRsity* (Keenan et al., 2013); therefore, all 100 loci were kept for subsequent analyses.

### 2.3 Population genetic analyses

#### 2.3.1 Genetic diversity of wild trout populations

For all wild brook trout sites, we calculated measures of genetic diversity, including inbreeding coefficient (*F*_IS_), observed and expected heterozygosity (*H*_o_ and *H*_e_), and effective population size (*N*_e_). Mean estimates of inbreeding and heterozygosity were calculated with 95% confidence intervals using the R package *diveRsity* (Keenan et al., 2013). *N*_e_ was calculated in NeEstimator v2 (Do et al., 2014) using an allele frequency cutoff (*P*‐crit) of 0.01, and estimates were calculated with corresponding confidence intervals adjusted using the jackknife method (Jones, Ovenden, & Wang, 2016). Significant differences between sites for diversity measures were determined based on nonoverlapping 95% confidence intervals.

#### 2.3.2 Population structure and genetic differences between wild and hatchery populations

Genetic structure was also examined among all wild brook trout sites as well as the hatchery samples. For the first comparison, a principal coordinate analysis (PCoA) was performed using the R package *adegenet* (Jombart, 2008). PCoA was performed with and without hatchery samples. In addition, isolation‐by‐distance (IBD) was also examined among wild brook trout populations. Least‐cost distance (km) was calculated between all sites as well as genetic divergence (Slatkin's linearized *F*_ST_) in Arlequin v3.5.2.2 (Excoffier & Lischer, 2010). To calculate least‐cost distances between sites, we used both the R package *marmap* (Pante & Simon‐Bouhet, 2013) and Google Earth. First, distance between river systems along the Nova Scotia shoreline (in the ocean) was calculated using the lc.dist function in *marmap*. Second, we measured distance to sites along the course of each river (approximate hydrologic distance) using Google Earth and these values were added to distance estimates. The relationship between genetic and geographic distance was examined using mantel.randtest function with 1,000 permutations in the R package *ade4* (Dray & Dufour, 2007).

For all subsequent analyses, hatchery samples were included in the analyses to compare genetic differences between hatchery and wild trout. Using Arlequin v3.5.2.2 (Excoffier & Lischer, 2010), pairwise genetic divergence (*F*_ST_) was calculated between all sites with corresponding significance (*p*‐values). To determine significant genetic divergence between sites, we used an adjusted (Bonferroni) alpha level of 0.0014 to account for multiple comparisons among the 35 sampling locations.

Bayesian clustering analysis was performed using STRUCTURE v2.3.4 (Pritchard, Stephens, & Donnelly, 2000) implemented through *parallelstructure* (Besnier & Glover, 2013) with three independent Markov chain Monte Carlo (MCMC) runs using 100,000 burn‐in and 500,000 iterations for each value of K (genetic clusters) ranging from 1 to 40. Although best K is often determined based on the ∆K statistic (Evanno, Regnaut, & Goudet, 2005), this statistic may be unreliable in complex evolutionary scenarios (Janes et al., 2017). Therefore, using STRUCTURE HARVESTER, we examined the plateau in mean LnPr(*X*|*K*) estimates to assess best K (Janes et al., 2017).

### 2.4 Assessing populations for evidence of hatchery introgression

To quantify levels of hatchery introgression, we first generated a simulated and centered hatchery population for the two hatcheries. These simulations were only performed for introgression analyses and not for analyses of genetic diversity and structure (above analyses). To generate these two simulated hatchery populations, we used freqbasedsim_AlleleSample function in the R package *hybriddetective* (Wringe, Stanley, Jeffery, Anderson, & Bradbury, 2017a). The purpose of these simulations was to generate a dataset that represents the average allele frequencies in the hatchery populations for each of the two hatcheries to reduce the presence of substructure that may bias assignment analyses.

Using simulated and centered datasets for both the Fraser's Mills and Margaree hatcheries, we next used two approaches to investigate evidence of introgression. First, STRUCTURE v2.3.4 (Pritchard et al., 2000) was used to assign individuals to genetic clusters (i.e., wild and hatchery clusters). This approach is similar to those described by Karlsson, Diserud, Moen, and Hindar (2014) where domestic populations are combined and centered to reduce biases associated with sample heterogeneity and different sample sizes, and to help standardize estimates of introgression across STRUCTURE runs (Karlsson et al., 2014). This centering approach has been applied by other studies to identify domestic introgression in wild populations (Sylvester et al., 2019; White et al., 2018; Wringe, Jeffery, et al., 2018). Ideally, wild populations would be centered as well; however, given the high levels of genetic structure found here (see Results) and unknown levels of hatchery introgression, we have not attempted to create a centered wild population. Instead, STRUCTURE was run separately for each river system as significant population structure was found within and among many river systems (see Results). Sites from the east branch of the Saint Mary's River system were also analyzed separately from those in the main branch. For each river system, the simulated Fraser's Mills hatchery population was included because records indicate that all stocked systems have been stocked with trout from Fraser's Mills hatchery at some point in time. Fraser's Mills samples were included in all analyses regardless of whether any stocking was present or absent in the river system. This is because Fraser's Mills has been stocked extensively across Nova Scotia in the past and thus may be useful for detecting introgression in the event that unreported stocking has occurred. The simulated Margaree hatchery population was also included in analyses if stocking records indicated that the strain had been previously stocked in that system (only sites on Cape Breton Island in eastern NS). We only included Margaree hatchery in these locations, because, unlike Fraser's Mills, these hatchery fish have not been extensively stocked at a large scale throughout the province and we do not expect that hatchery fish will move large distances (Gowan & Fausch, 1996; Hartman & Logan, 2010; Moore et al., 1985). For analyses within each river system, a location prior was used for the simulated hatchery population(s) as well as for the uppermost site in the watershed. We included the location prior for the uppermost site to represent a “pure” wild location because we expected this site to have more limited hatchery introgression, as it should be more difficult to access the upper reaches of the watershed. For the three rivers where no upper site was present (Upper Medway, Salmon River, and St. Mary's Bay), no location prior was used for the wild location. STRUCTURE v2.3.4 (Pritchard et al., 2000) was executed through *parallelstructure* (Besnier & Glover, 2013) with three independent Markov chain Monte Carlo (MCMC) runs using 100,000 burn‐in and 500,000 iterations for each value of *K* ranging from 1 to the total number of wild and hatchery sites present in the analysis. Output plots were visually inspected for best *K*, which was dependent on population structure within the watershed and how many hatchery populations were included. Using the best K (range *K* = 2–4), CLUMPP (Jakobsson & Rosenberg, 2007) was used to determine the mean proportion of hatchery membership (*Q*‐value) across runs for each wild site. In cases where two hatchery sources were used, the mean membership proportions to each hatchery source were summed to determine total hatchery membership. Proportion of hatchery membership for each site was considered a measure of hatchery introgression for the site.

Next, we used NEWHYBRIDS v1.1 (Anderson, 2008) to assign individuals within each site to one of six possible genotype classes, which included pure wild, pure hatchery, *F*_1_ hybrid, *F*_2_ hybrid, backcross‐wild, or backcross‐hatchery. Given that population structure was found within most river systems (see Results), analyses were performed for each site (within rivers) separately so that there was only one hatchery and one wild population in each NEWHYBRIDS run. As above, simulated Fraser's Mills samples were used in all analyses. In locations where both Margaree and Fraser's Mills trout had been stocked, analyses were preformed separately for each hatchery source. Datasets for each site were run in NEWHYBRIDS in parallel through the R package *parallelnewhybrid* (Wringe, Stanley, Jeffery, Anderson, & Bradbury, 2017b) using 100,000 burn‐in and 500,000 MCMC iterations. For each individual, the genotype class with the highest posterior probability was used to classify each individual at each site. For some sites, NEWHYBRIDS runs failed to converge, suggesting that loci did not have enough discriminatory power to differentiate between the wild and hatchery individuals. Therefore, we only report results for sites where the wild and hatchery population could clearly be discriminated, and for these sites, we calculated the proportion of individuals assigned to each genotype class.

### 2.5 Environmental and anthropogenic correlates to genetic structure and introgression

#### 2.5.1 Anthropogenic variables: Hatchery stocking and human disturbance

Detailed hatchery stocking records were obtained from the province of Nova Scotia (source: https://data.novascotia.ca/Fishing-and-Aquaculture/Nova-Scotia-Fish-Hatchery-Stocking-Records/8e4a-m6fw/data). Records begin in 1976, and stocking information includes number and size of stocked fish, location, date, stocking objective (spring vs. fall program), and hatchery/strain origin. We compiled brook trout stocking data for all water bodies within the 12 systems sampled in our study. For each river system, we calculated the total number of fish stocked, number of stocking events, mean number and weight (grams) of fish stocked per event, number of fish stocked in fall and spring programs, proportion of adults stocked, and the first and last year in records (see Table 2 for stocking details by river system). Stocking did not occur every year in every system (see Figure S2 for number of fish stocked each year), and thus, we also calculated the number of years in which stocking events occurred (Table 2). Mean year of stocking events was also calculated across all stocking events (Table 2). In addition, given that the intensity of stocking may differ for different locations within the rivers (Colautti, 2005), we also estimated a measure of propagule pressure for each sampling site in our study. Propagule pressure can be calculated (Keyser et al., 2018) based on the number of fish stocked during each stocking event and the distance between a given site and the stocking site:

$$Propagule\;pressure\;for\;a\;given\;site\;in\;a\;river\;\left(R\right)=\sum_{i,d=1}^{S}\frac{F_{i,d}}{LCD(S_{i,d}\;to\;R)}$$

where *R* is a given sampling site, *F_i,_*_d_ is the number of fish stocked at a given location (*S_i,d_*) on a given date, and *LCD* is the least‐cost distance between the sampling site *R* and the stocking site *S_i,d_*. For each site, *LCD* was calculated based on the straight‐line distance (kilometers) between the sampling site and the nearest stocking site within that river. Following this equation, total propagule pressure is based on the sum of propagule pressure from all stocking events.

In addition to stocking data, we also calculated the average human density (persons/km^2^) around each site. Human population density (resolution 0.1 degrees; source: https://neo.sci.gsfc.nasa.gov/) was used as a proxy for habitat disturbance (see Lehnert, Kess, et al., 2019), and density values were averaged from a larger grid (0.5**°** latitude × 0.5**°** longitude) around each site to capture human activity in the surrounding region.

**Table 2:** Summary of brook trout (*Salvelinus fontinalis*) stocking data for river systems based on provincial records

River system	Total number stocked	% Adult	Stocking events	Mean no. stocked	Mean weight (g)	Fall stocked	Spring stocked	Years			
								First record	Last record	Years with stocking	Mean year
St. Mary's Bay	0	–	–	–	–	0	0	–	–	0	–
Annapolis	669,367	20.2	265	2,526	89.6	178,587	490,780	1976	2016	38	1995
Upper Medway	1,150,151	19.6	488	2,357	267.33	575,666	574,485	1976	2016	34	1995
Cornwallis	101,070	61.2	96	1,053	131.64	47,510	53,560	1976	2016	35	1991
LaHave	781,752	14.2	269	2,906	94.39	389,985	391,767	1976	2016	33	1996
Musquodoboit	249,223	27.6	130	1,917	95.59	158,511	90,712	1978	2018	38	2000
Salmon	0	–	–	–	–	0	0	–	–	0	–
East River Pictou	543,651	4.7	166	3,275	63.68	268,019	275,632	1976	2018	39	1996
St. Mary's River	0	–	–	–	–	0	0	–	–	0	–
River Denys	5,457	44.6	3	1,819	53.17	3,025	2,432	1976	1999	3	1985
Margaree	582,710	5.7	165	3,532	205.2	321,850	260,860	1976	2018	37	2000
Baddeck	110,686	35.6	55	2,012	290.5	17,294	93,392	1976	2016	19	1996

Note

Information includes total number of fish stocked overall, number (and percentage) of fish stocked that were adults (≥1 year from hatch), total number of stocking events, mean number of fish stocked per event, mean weight (grams) of fish stocked per event, number of fish stocked in fall and spring programs over all years, and the first and last year in records. In addition, the number of years in which stocking events occurred and the mean year of stocking events were calculated.

#### 2.5.2 Environmental variables

Environmental data were also compiled for each sampling site. Based on latitude and longitude of each site, we extracted 19 bioclimatic variables representing precipitation and temperature data from WorldClim version 2.0 (http://worldclim.org/version2) at a spatial resolution of 2.5 arc minutes (Fick & Hijmans, 2017). Elevation (meters) was also extracted for each site using topography data from *marmap* R package (Pante & Simon‐Bouhet, 2013).

#### 2.5.3 Environmental and anthropogenic associations with genetic structure

Distance‐based redundancy analysis (dbRDA) was performed using the R package *vegan* (Oksanen et al., 2017) with environmental and anthropogenic variables as constraining factors to explain genetic variation among wild brook trout sites. For the genetic data, we performed a PCoA using a distance matrix generated based on allele frequencies and we retained the first two PCo axes for the response in the dbRDA. PCo axis values were averaged among all individuals at a site to generate population‐level values. For the dbRDA, provided that many of the 19 bioclimatic variables are highly correlated with each other, these variables were collapsed into principal components (PCs) using prcomp function in R. Similarly, stocking variables were also highly correlated with each other, and thus, stocking data were also collapsed into PCs. For the stocking PCA, we included 8 variables, which were propagule pressure, total number of fish stocked, number of fish stocked in fall and spring, proportion of adult fish stocked overall, mean number and weight of fish per stocking event, and number of years since mean year of stocking. The number of years since mean year of stocking was determined by subtracting the value from 2018, and all sites without stocking were given a value of 42 years (no stocking record since 1976). Prior to PCAs for bioclimatic and stocking data, all variables were scaled using the scale function in R. In the dbRDA, we included all PC axes that explained >80% of the variance. Human density, elevation, and geographic distance from the westernmost site were also scaled and included as additional variables in the dbRDA. Geographic distance was calculated as distance by water (km) from Duffy Brook in Saint Mary's Bay (westernmost site). Partial dbRDA was then performed with the same variables after controlling for geography (hydrologic distance from the westernmost site). The significance of each variable was determined by assessing the marginal effects of each term in the model with all other variables using 1,000 permutations with the ANOVA function in *vegan.*

#### 2.5.4 Environmental and anthropogenic associations with hatchery introgression

In addition to determining how environmental and anthropogenic variables correlate with genetic structure, we also used multiple regression to investigate whether factors such as environmental and anthropogenic variables (same as above) were associated with the level of hatchery introgression. The environmental variables used in the model included principal components of bioclimatic variables (enviro‐PCs 1–3; see above) as well as elevation. Anthropogenic variables included stocking principal components (stocking PCs 1–2) and human population density. The full model included these seven variables, and variance inflation factors were all below 5 (max value 4.28), suggesting generally low collinearity among variables. The level of introgression was determined by the proportion of hatchery membership (*Q*‐value from STRUCTURE analysis) for each site and was log‐transformed and used as the response in the model.

## 3 RESULTS

### 3.1 Population genetic analyses

#### 3.1.1 Genetic diversity of wild trout populations

Estimates of genetic diversity in wild trout populations are presented in Table 1 and Figure S3. Mean heterozygosity estimates varied across sites, where mean *H*_o_ and *H*_e_ across all sites were both 0.36. Heterozygosity estimates (*H*_o_ and *H*_e_) were generally higher for sites in the eastern part of the province (Cape Breton Island), where Angus Farquhars Brook (Ang) in Baddeck River had the highest expected heterozygosity estimates (*H*_e_ = 0.40), but the majority of sites in the province (all except two sites) did not differ significantly in heterozygosity from Angus Farquhars Brook. Only one site had significantly lower observed heterozygosity relative to all other sites, and this was Greens Brook (GR) in Saint Mary's River (*H*_o_ = 0.22; *H*_e_ = 0.22).

Observed and expected heterozygosity were similar to each other within most sites, as inbreeding coefficients (*F*_IS_) suggested no significant deviations from random mating in the majority of sites. Only two sites showed significant evidence of inbreeding based on bootstrapped 95% confidence intervals, and this included the Upper Medway (UM) system and Cooks Brook (CO) in the LaHave system (Table 1; Figure S3). Two sites in Saint Mary's River (Kelly Brook [Kel] and Clarks Creek [Cla]) had negative *F*_IS_ values that deviated significantly from zero indicating an excess of heterozygotes in the population.

In addition to higher heterozygosity estimates in the eastern part of the province, effective population size (*N*_e_) was also generally higher in this part of the province. Harris Brook (HA) in the Baddeck River had the largest positive *N*_e_ (*N*_e_ [95% CI] = 3,188.5 [581.3‐Inf]), although two other sites had negative *N*_e_ values, which can be indicative of large population sizes (Sawmill Site [SA] *N*_e_ = −1,729.3 [2,061.6‐Inf]; Clarks Creek [Cla] *N*_e_ = −774.1 [402.3‐Inf]). The site with the smallest *N*_e_ was Glencoe Brook (GL) in East River Pictou (*N*_e_ = 10.1 [9.2–11.1]).

#### 3.1.2 Population structure and genetic differences between wild and hatchery populations

**Figure 2:**
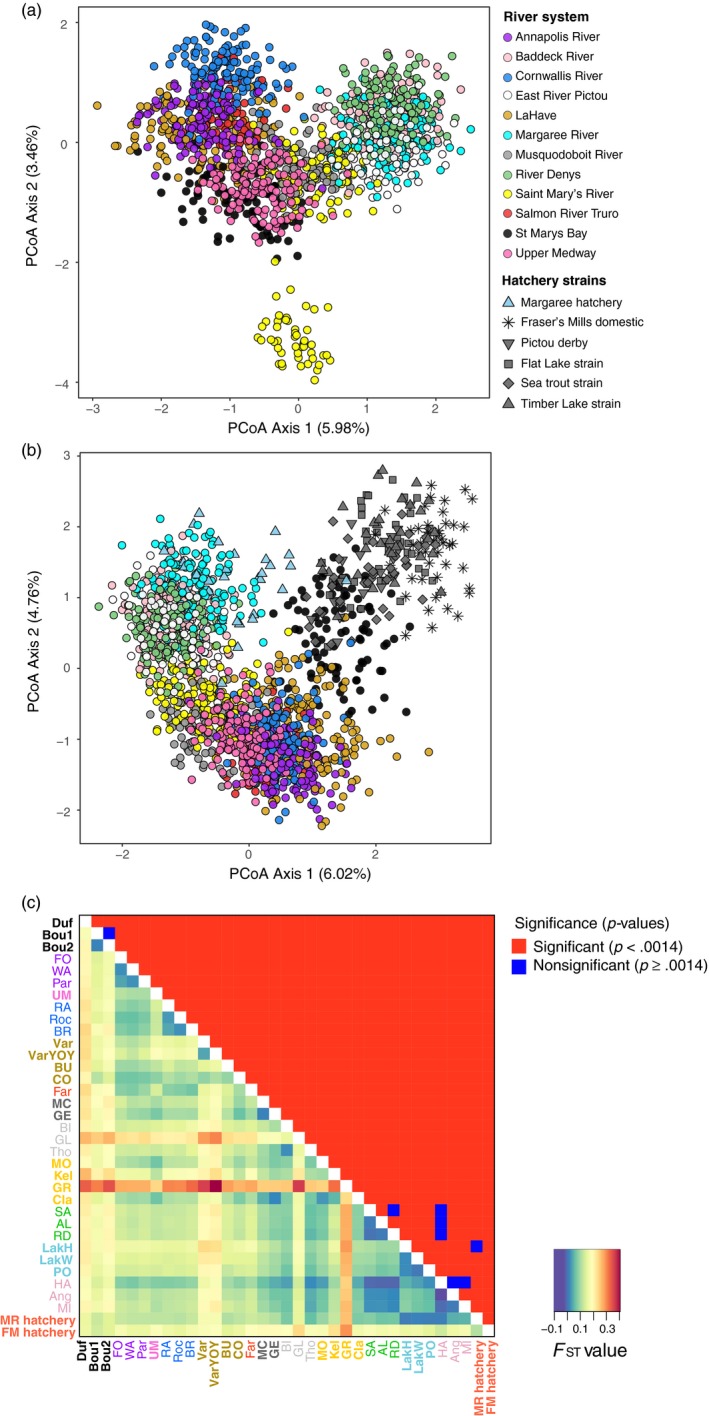
Genetic relationships among brook trout (*Salvelinus fontinalis*) populations in Nova Scotia based on 100 microsatellites. (a) Principal coordinate analysis (PCoA) of wild brook trout with each data point (individual) colored by river system and (b) PCoA with both wild and hatchery brook trout. (c) Pairwise genetic divergence (*F*_ST_; lower triangle) and associated significance (*p*‐values; upper triangle) between all populations (wild and hatchery). All pairwise *F*_ST_ values are provided in Table S3. Population names are colored and arranged by river system from west to east

For wild populations, principal coordinate analysis (PCoA) separated individuals from sites in the eastern and western part of the province along the first PCo axis (Figure 2a). Intermediate sites included Musquodoboit River and Saint Mary's River that are located in the central part of this range. Along the second PCo axis, one site within the Saint Mary's River (Greens Brook [GR]) was separated from all other sites and this site was characterized by significantly lower diversity relative to other sites (see above). Overall, genetic structuring of wild brook trout populations was consistent with significant isolation‐by‐distance (*p* = .001; *r* = .23; Figure S4).

Using both hatchery and wild samples in the PCoA (Figure 2b), hatchery samples from Margaree hatchery clustered with individuals from Margaree River, whereas samples from Fraser's Mills hatchery were separated from most populations with the exception of St. Mary's Bay. To further investigate the close association between Fraser's Mills and St. Mary's Bay, a separate PCoA was performed on only these samples and revealed clearly distinct clusters between hatchery and wild individuals on the first PCo axis (Figure S5).

Estimates of genetic divergence (*F*_ST_) between sites were significant for the majority of pairwise comparisons, including sites within the same river system (Figure 2c and Table S3). Only a few comparisons within river systems were not significantly divergent, and this included sites in St. Mary's Bay (Boudreau Brook sites 1 and 2; *F*_ST_ = 0.01), sites in River Denys (Sawmill Site [SA] and River Denys sites [RD]; *F*_ST_ = 0.0005), and sites in Baddeck (Harris Brook [HA] and Angus Farquhars Brook [Ang], *F*_ST_ = −0.027; Harris Brook [HA] and Mill Brook [MI], *F*_ST_ = −0.0045). All sites were significantly divergent from sites in other river systems except for one site in Baddeck (Harris Brook; HA), which was not significantly divergent from all sites in the River Denys systems (*F*_ST_ < −0.007). Low genetic divergence between River Denys and Baddeck sites could indicate migratory anadromous trout, as some of these locations are in lower reaches of the systems. Both hatchery samples (Fraser's Mills and Margaree) were significantly genetically divergent from most wild brook trout sampling sites, with one exception. The Margaree hatchery was not genetically divergent from samples that had evidence of fin erosion (potential hatchery origin) in Lake O'Law Brook in Margaree River (*F*_ST_ = 0.007). Samples without fin erosion from the same location were genetically divergent from the Margaree hatchery (*F*_ST_ = 0.01). Fraser's Mills was significantly divergent from all wild sites. Although Fraser's Mills hatchery strains were combined for these analyses, genetic divergence (*F*_ST_) between the hatchery strains and sample sizes are provided in Table S4.

**Figure 3:**
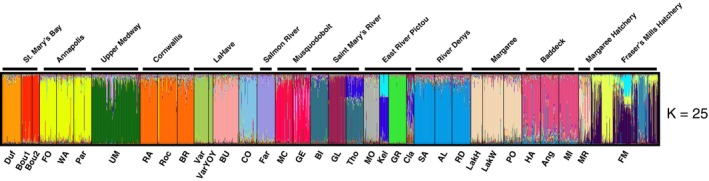
Results of Bayesian clustering analysis in STRUCTURE for brook trout (*Salvelinus fontinalis*) sampling locations across Nova Scotia. STRUCTURE plots show individual assignments to *K* = 25 genetic clusters, which were determined as the optimal number of K based on the plateau in mean LnPr(*X*|*K*) estimates (see Figure S6)

Bayesian clustering analyses revealed evidence of genetic structure across the province where best *K* was 25 genetic clusters (see Figure S6), which separated all river systems from each other and revealed substructure within some systems (Figure 3). Using *K* = 25, hatchery samples from Margaree clustered with wild sites in the Margaree River, whereas the Fraser's Mills Hatchery had membership to several clusters that were distinct from wild samples.

### 3.2 Assessing populations for evidence of hatchery introgression

**Figure 4:**
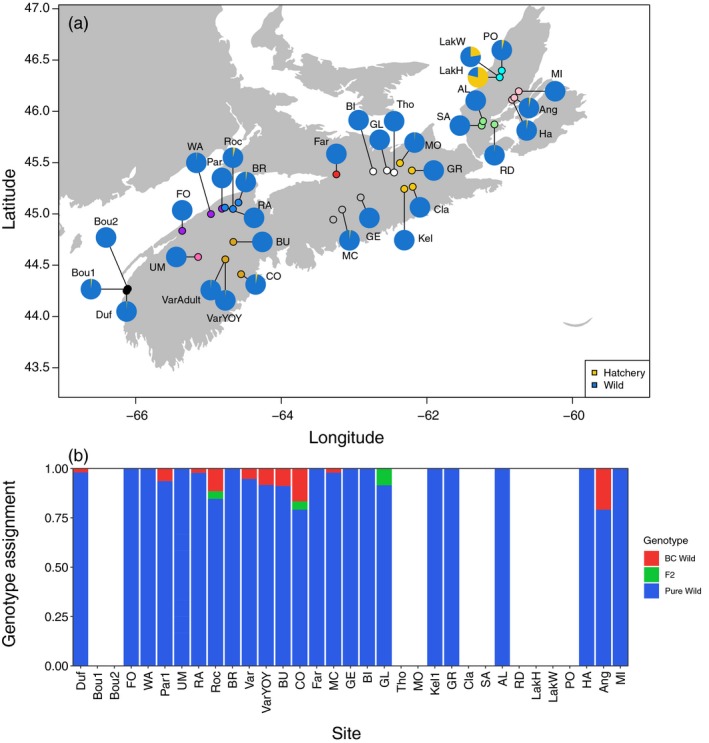
(a) Map and pie charts indicating the proportion hatchery introgression (yellow) for brook trout (*Salvelinus fontinalis*) populations calculated based on population‐specific membership (*Q*‐value) to the hatchery cluster in STRUCTURE analyses by river system. (b) Proportion of individuals assigned to pure and hybrid genotype classes calculated in NEWHYBRIDS. A total of six possible genotype classes (pure wild, pure hatchery, *F*_1_, *F*_2_, backcross‐wild, and backcross‐hatchery) were included in the analysis. Populations with missing data indicate that analysis did not converge, suggesting reduced discriminatory power between hatchery and wild populations due to genetic similarities

To assess introgression, STRUCTURE runs were performed within each river system with centered hatchery populations (see Methods). Evidence of hatchery introgression was generally low across the province. Based on population‐specific membership to the hatchery cluster, all sites had less than 5% hatchery ancestry with the exception of Lake O'Law Brook in Margaree (Figure 4a). Two groups of samples were genotyped from Lake O'Law, which included samples with and without fin erosion. Samples with fin erosion (suspected of being hatchery origin) had high membership to the hatchery cluster (mean *Q*‐value = 0.78), whereas samples without fin erosion showed moderate levels of hatchery membership (mean *Q*‐value = 0.23). Following Harbicht et al. (2014), we considered trout to be pure hatchery or pure wild if membership to the hatchery cluster was greater than 90% (*Q*‐value > 0.90) or less than 10% (*Q*‐value < 0.10), respectively. For Lake O'Law samples with fin erosion, 52% (*n* = 16/31) were pure hatchery trout and only two trout were pure wild origin. Conversely, for samples without fin erosion, 69% (*n* = 38/55) were pure wild trout and only three trout were pure hatchery. Overall, aside from Lake O'Law, hatchery introgression was low in wild populations.

Using NEWHYBRIDS, we found similarly low levels of hatchery introgression across sites (Figure 4b), as no recent hybrid individuals (*i.e.*, no *F*_1s_) or pure hatchery trout were found at wild sites. While not all sites could be examined using NEWHYBRIDS given reduced discriminatory power of loci in some locations, we found that sites were primarily (>75%) comprised of pure wild trout with evidence of potential *F*_2_ hybrids and backcross‐wild individuals at some sites (Figure 4b). Reduced discriminatory power for some locations is not unexpected, as previous work using NEWHYBRIDS indicates that when domestic individuals are derived from local sources, as is the case for Margaree, hundreds of loci can be required to accurately identify hybrids and pure individuals in the wild (Wringe, Anderson, Jeffery, Stanley, & Bradbury, 2018). We note that analyses using Margaree River hatchery did not converge, and thus results represent potential hybridization associated with the Fraser's Mills hatchery only.

### 3.3 Environmental and anthropogenic correlates to genetic structure and introgression

#### 3.3.1 Environmental and anthropogenic associations with genetic structure

**Figure 5:**
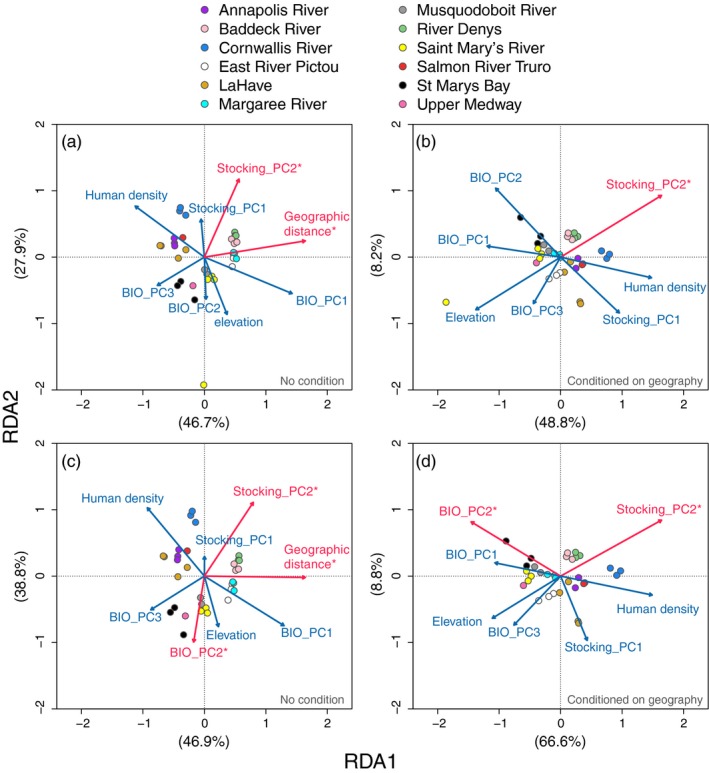
Genetic structure of brook trout (*Salvelinus fontinalis*) populations and associations with environmental (bioclimatic variables and elevation) and anthropogenic (stocking and human density) variables based on redundancy analysis (RDA). Panels (a‐b) include all wild populations where RDAs were performed without (a) and with (b) geography as a conditional variable. Panels (c–d) include all wild populations except GR (genetically divergent) where RDAs were performed without (c) and with (d) geography as a conditional variable. Red text and asterisk associated with arrows indicate a significant association with genetic variation. Percent of variance explained by each RDA axis is provided in parentheses for each panel

For distance‐based redundancy analysis (dbRDA), bioclimatic and stocking data were collapsed into principal components (PCs) as many showed high correlations with each other (see Tables S5 and S6). The 19 bioclimatic variables were collapsed into PCs, and the first three PCs were retained as they explained 81% of the variation (see Figures S7 and S8 for variable contributions and loadings). The bioclimatic PC axes 1, 2, and 3 explained 39.0%, 29.8%, and 11.9% of the variance, respectively. Similarly, stocking data were also collapsed into PCs, and the first two axes were retained and explained 83% of the variation in stocking (see Figures S9 and S10 for variable contributions and loadings). The stocking PC axes 1 and 2 explained 62.7% and 20.1% of the variance, respectively. dbRDA revealed significant effect of geographic distance (*p* = .001) on the first RDA axis and stocking (PC2; *p* = .006) on the second RDA axis related to genetic structure of brook trout populations (Figure 5a). After controlling for geography (hydrologic distance from the westernmost site), partial dbRDA revealed that the anthropogenic variable of stocking PC2 significantly explained genetic structure of wild brook trout (Figure 5b). In this partial dbRDA, RDA axes 1 and 2 explained 61% of the genetic variation (overall $R_{\text{adj}}^{2}$ = 0.30). The stocking variable contributing to stocking PC2 is the proportion of adults stocked in a system, and this variable showed a positive relationship with genetic population structure associated with PCo axis 2 that separated populations along RDA2 (see Figure S11a).

Notably, one site (Greens Brook [GR] in Saint Mary's River) in our analyses was divergent from all other sites, and thus, we performed the same analyses as above with this site excluded in case this one site skewed our analyses. Without Greens Brook (GR), dbRDA revealed a significant effect of geography (hydrologic distance from the westernmost site) (*p* = .001), stocking PC2 (*p* = .001), and bioclimatic PC2 (*p* = .001) on genetic structure of brook trout (Figure 5c). After controlling for geography (geographic distance), partial dbRDA revealed significant effect of bioclimatic PC2 (*p* = .005) and stocking PC2 (*p* = .004) on brook trout genetic structure (Figure 5d). Overall, in the partial dbRDA, RDA axes 1 and 2 explained 78% of the genetic variation (overall $R_{\text{adj}}^{2}$ = 0.43). The significant variables (bioclimatic PC2 and stocking PC2) tended to be associated with population structure along the second axis (PCo axis 2). Consistent with the first RDA (with all sites), stocking PC2 (i.e., proportion of adults stocked) showed a positive relationship with genetic population structure (PCo axis 2) when GR was removed (see Figure S11b). Bioclimatic variables loading the highest on bioclimatic PC2 included BIO4 and BIO7 representing measures of temperature variability, including temperature seasonality and annual temperature range, respectively. Both bioclimatic variables showed a positive relationship with population structure based on PCo axis 2 (Figure S11c,d).

#### 3.3.2 Environmental and anthropogenic associations with hatchery introgression

Multiple regression analysis revealed that no anthropogenic or environmental variables significantly explained levels of hatchery introgression in wild brook trout populations (Table 3; *F*_7,25_ = 1.89, *p* = .12; $R_{\text{adj}}^{2}$ = 0.16). Nonetheless, we note that elevation approached statistical significance in the model (*p*‐value = .054). However, given that sites in Lake O'Law had much higher levels of introgression relative to all other sites, we removed Lake O'Law and reran the analysis. Re‐analysis again revealed that no variables significantly explained levels of hatchery introgression in wild brook trout populations (Table 3; *F*_7,23_ = 1.36, *p* = .27; $R_{\text{adj}}^{2}$ = 0.08).

**Table 3:** Multiple regression results for variables explaining levels of hatchery introgression in wild brook trout (*Salvelinus fontinalis*) populations in Nova Scotia. Multiple regressions were performed (A) with and (B) without samples from Lake O'Law in Margaree where high levels of hatchery ancestry were found.

(A)	Variable	Estimate	*SE*	*t* value	*p*
	(Intercept)	−1.968	0.097	−20.24	<.001
	Bioclim_PC1	0.097	0.075	1.29	.21
	Bioclim_PC2	0.032	0.050	0.63	.54
	Bioclim_PC3	−0.074	0.075	−0.99	.33
	Stocking_PC1	0.083	0.048	1.73	.10
	Stocking_PC2	−0.058	0.100	−0.58	.57
	Human density	0.158	0.193	0.82	.42
	Elevation	−0.294	0.145	−2.03	.05
		*F*_7,25_ = 1.88	*p* = .12	$R_{\text{adj}}^{2}$ = 0.17	

(B)	Variable	Estimate	*SE*	*t* value	*p*
	(Intercept)	−2.067	0.081	−25.59	<.001
	Bioclim_PC1	0.082	0.060	1.38	.182
	Bioclim_PC2	0.029	0.040	0.74	.468
	Bioclim_PC3	0.029	0.065	0.45	.660
	Stocking_PC1	0.018	0.041	0.45	.658
	Stocking_PC2	0.002	0.080	0.02	.984
	Human density	0.234	0.154	1.52	.142
	Elevation	−0.203	0.117	−1.74	.096
		*F*_7,23_ = 1.36	*p* = .27	$R_{\text{adj}}^{2}$ = 0.08	

Note

Table includes coefficient estimates with standard errors (*SE*), *t* value, and significances (*p*‐value).

## 4 DISCUSSION

Animal populations worldwide are threatened with losses (Burkhead, 2012; Ceballos et al., 2017), and increasingly, supplementation of captive‐bred individuals is being used to help support many species (Ebenhard, 1995; Naish et al., 2007; Seddon et al., 2007). Salmonid species represent a heavily propagated group of animals (Araki et al., 2008; Hilborn, 2007; Lackey et al., 2006) within which the negative consequences of releasing captive‐bred individuals have been documented in wild populations (Araki & Schmid, 2010; Brannon et al., 2004; Naish et al., 2007). Specifically, genetic interactions between hatchery and wild salmonids may present long‐lasting threats to wild populations (Muhlfeld et al., 2017; Naish et al., 2007; Willoughby & Christie, 2019), and thus, it is important to identify and monitor such interactions between hatchery fish and local populations. In Nova Scotia, Canada, brook trout is the primary sport fish, but recreational catches of brook trout have declined by approximately 60% in recent decades (Nova Scotia Department of Agriculture & Fisheries, 2005). To meet angling needs, extensive hatchery stocking has occurred in the province. Therefore, this brook trout system provided an opportunity to investigate the impact of extensive hatchery stocking programs on the genetic structure of wild trout populations and investigate factors that influence hatchery introgression.

In our study, we characterized genetic structure and hatchery introgression in wild brook trout populations from 12 river systems across Nova Scotia. We found extensive genetic structure among wild brook trout populations and limited evidence of hatchery introgression despite decades of stocking efforts. Only one sampling location, Lake O'Law Brook in the Margaree River, had a substantial proportion of hatchery‐derived brook trout, whereas in all other populations, the levels of hatchery introgression were less than 5%. Further, the amount of hatchery stocking in a watershed was not significantly associated with levels of hatchery introgression, suggesting that extensive stocking efforts have not had substantial genetic impacts on wild trout populations.

Despite records of over 23 million hatchery brook trout released in the province in the last four decades, our results suggest that hatchery fish are not successfully reproducing in the wild. There are at least three potential explanations for these results. First, stocked hatchery trout may be successfully harvested after release if hatchery trout are more prone to angling mortality (García‐Marín, Sanz, & Pla, 1999; Hunt, 1979; Mezzera & Largiader, 2001). Behavioral studies have found that hatchery‐reared salmonids can be more bold and aggressive and exhibit greater risk‐taking behavior than wild conspecifics (Deverill, Adams, & Bean, 1999; Sundström et al., 2004; Swain & Riddell, 1990; Vincent, 1960), which may make them more susceptible to angling pressure. A second possibility is that hatchery trout are not surviving in the wild after release, as it is not uncommon for captive‐bred animals to have lower survival in the wild (Jackson, Schuster, & Arcese, 2016; Rantanen, Buner, Riordan, Sotherton, & Macdonald, 2010). Lower survival of hatchery relative to wild trout has been documented under natural conditions (Danzmann & Ihssen, 1995; Flick & Webster, 1964; Miller, Close, & Kapuscinski, 2004; Miller, 1954) potentially due to reduced foraging ability (Ersbak & Haase, 1983), greater predation risk (Sundström et al., 2004; Vincent, 1960), and lower swimming performance (Pedersen, Koed, & Malte, 2008). Further, hatchery trout may not be well adapted to local environmental conditions (Jones et al., 1996) given the genetic, phenotypic, and behavioral changes that can result from captive‐breeding programs. Finally, a third potential hypothesis explaining limited introgression is that hatchery trout do survive to reproduction but are unable to successfully interbreed with wild conspecifics due to pre‐ or postzygotic isolating mechanisms. Hatchery fish can have reduced reproductive success relative to wild individuals (Araki, Ardren, Olsen, Cooper, & Blouin, 2007; Christie, Ford, & Blouin, 2014; Ford, Murdoch, Hughes, Seamons, & LaHood, 2016; Miller, Ward, & Schreiner, 2014), which may be attributed to changes in mating behavior (Berejikian et al., 2001; Fleming & Gross, 1993; Schroder et al., 2008). Even if mating does occur, genetic differences between hatchery and wild populations could lead to nonviable offspring or hybrid offspring that are maladapted to the local environment (Mavarez, Audet, & Bernatchez, 2009). Any or all of these potential hypotheses may explain the low levels of hatchery introgression found in our study.

In animal captive‐breeding programs, the goal of many programs is to reduce the amount of generations in captivity (Williams & Hoffman, 2009). By reducing the amount of time spent in captivity, this can reduce the potential for selection (intentional or unintentional) and thus limit the amount of genetic change that occurs in the captive population (Waters et al., 2018, 2015; Williams & Hoffman, 2009). In our study, only one location in the Margaree River (Lake O'Law Brook) showed high numbers of hatchery‐derived trout based on membership coefficients from STRUCTURE (Harbicht et al., 2014). The high number of hatchery trout found in the Margaree system is notable given that this is the only river system where stocking occurs with a locally derived strain that incorporates wild broodstock. Lower levels of genetic divergence between Margaree hatchery and wild trout (*F*_ST_ ≤ 0.01) may thus explain why rates of introgression and hatchery survival are higher as the locally derived strain and wild sourced individuals may be better adapted to the environment compared to non‐native strains. In addition, at the Lake O'Law site, samples were collected in 2017, and recent stocking efforts in 2016 in the Margaree system (*n* = 94,475 stocked trout) may have contributed to the large number of hatchery fish collected in Lake O'Law Brook. However, a nearby upstream site, Portree Brook, contained only a single hatchery trout and over 81% pure wild trout despite comparable proximity to stocking sites. Portree Brook was sampled in 2018; thus, it is possible that higher numbers of hatchery trout may have been found at this location in the previous year, dispersal of hatchery trout is limited to certain regions of the watershed, or this location is less accessible to stocking.

In contrast to Margaree hatchery, the Fraser's Mills hatchery population was an order of magnitude higher in genetic divergence from all wild sites (*F*_ST_ ≥ 0.127), and all sites where Fraser's Mills were stocked had low levels of introgression. The exact genetic origin of Fraser's Mills trout is unknown, but several strains at the hatchery appear to be genetically distinct from each other (see Table S4). Further, some strains may be derived from trout populations outside of Nova Scotia (Department of Marine & Fisheries, 1929), and this may result in trout that are maladapted to local rivers. Our genetic data showed that Fraser's Mills trout grouped most closely with trout from St. Mary's Bay; however, hatchery samples were still genetically distinct from these wild sites (*F*_ST_ ≥ 0.130; see Figure S5). There are no provincial records of stocking in St. Mary's Bay, which could suggest that some hatchery strains were originally derived from St. Mary's Bay (or a closely related system) or that undocumented stocking has previously occurred. Future work focused on mitochondrial DNA will help identify the genetic origins of hatchery trout.

In many captive‐breeding programs, the goal of stocking animals in the wild is to increase the number of individuals in the wild to conserve and promote a self‐sustaining population (Williams & Hoffman, 2009), and in salmonid stocking programs, the goals can relate to species conservation and/or to increase harvest. Therefore, the interpretation of the success of Nova Scotia's current brook trout stocking program is dependent on the goals of the program. If anglers are capturing the majority of stocked trout after release, then stocking is successfully supporting the recreational fishery and reducing harvest pressure on wild trout. Few studies have investigated what proportion of recreational catches are hatchery and wild origin in Nova Scotia (Alexander, 1975), but higher exploitation rates of hatchery trout (Hunt, 1979) could explain in part the results observed in our study. Consequently, our results suggest that hatchery stocking may not have a direct impact on the genetic integrity of wild trout, although we acknowledge that with our current data, we cannot evaluate how hatchery stocking may be influencing wild populations through mechanisms other than genetic introgression (e.g., competition, disease transfer). 

Given the limited hatchery introgression, the genetic characterization of wild brook trout populations in our study can serve as a genetic baseline for the current status of wild brook trout in the province that can be used to assess changes in the future. Our results demonstrate that populations are highly structured with significant effect of isolation‐by‐distance (IBD). High levels of genetic structure are not unexpected, as brook trout ranks among the most highly structured animal species (Ward et al., 1994), and recent work has demonstrated this strong interpopulation differentiation in the species (Ferchaud et al., 2019). Genetic differentiation among wild populations was associated with environmental variation including temperature variability as well as the proportion of adults stocked in a river. Temperature variability (annual range and temperature seasonality) was associated with brook trout genetic structure (PCo axis 2), consistent with other genetic studies in the species (Kanno, Vokoun, & Letcher, 2011). Thermal habitat is important to brook trout populations, as the species generally prefers cooler water temperatures, and demographic changes in brook trout populations have been associated with warming water conditions (Stranko et al., 2008). We also found an association between the proportion of adults stocked in a river and genetic structure (PCo axis 2). This association is unclear, as this stocking variable was not associated with introgression level, and rivers with higher proportion of adult stocking (i.e., Cornwallis, River Denys, and Baddeck) also tended to have lower levels of stocking overall (*r *= −.20; see Table 2 and Table S6), suggesting that this cannot be attributed to hatchery introgression in these locations. Genetic structure along PCo axis 2 (see Figure 2a) does not reflect geography but may relate to genetic diversity of populations and therefore could instead reflect life history variation in the province or associations with unidentified environmental variation. More data are needed to better understand this potentially coincidental relationship.

In our study, patterns of genetic diversity varied across sites, where sites in the eastern part of the province (Cape Breton Island) generally showed higher (but not significant) levels of diversity. Some of these locations may harbor migratory anadromous trout, which may contribute to increased genetic diversity in the region (Ferchaud et al., 2019). The majority of sites were estimated to have effective population sizes (*N*_e_) greater than 50, and only one site may be of particular concern given its low *N*_e_ estimate. *N*_e_ for Glencoe Brook (GL in East River Pictou) was 10.1 (95% CI: 9.2–11.1), and *N*_e_ estimates below 50 individuals may be a concern because, in small populations, the effects of genetic drift will outweigh the influence of selection making it difficult for such populations to respond to environmental change (Charlesworth, 2009). Temporal genetic monitoring of such populations may be a useful approach for evaluating future changes at sites of concern (Schwartz, Luikart, & Waples, 2007).

In conclusion, our study found that hatchery introgression is limited in Nova Scotia brook trout, with the exception of one location where a local strain is used for hatchery stocking. We acknowledge that the limited hatchery introgression found here made it difficult to address one of our goals of identifying environmental and anthropogenic variables that influence levels of hatchery introgression. While other studies have found evidence of hatchery introgression in brook trout populations, our results are comparable to other studies conducted in riverine systems where limited introgression is often found (Jones et al., 1996; Kazyak et al., 2018; Kelson et al., 2015; White et al., 2018). Further, although our study primarily detected low levels of introgression, it is possible that introgression may be limited to a few genomic regions (Bay, Taylor, & Schluter, 2019; Lehnert, Bentzen, et al., 2019; Ozerov et al., 2016) but these regions cannot be resolved with the current panel of microsatellite markers. Evidence of introgression of hatchery alleles associated with ecologically relevant traits, such as growth, has been documented in other brook trout studies (Lamaze et al., 2012), and thus, future studies that identify tracts of hatchery ancestry in the genome of wild individuals could help determine how and whether previous introgression events have influenced these wild trout populations. Nonetheless, our results suggest that the majority of stocking efforts in Nova Scotia may have limited direct genetic effects on wild populations; however, to ensure the protection of wild brook trout in the future, studies are needed to investigate how the presence and abundance of hatchery trout may be impacting wild brook trout populations through ecological interactions (i.e., indirect genetic effects). In addition, the high levels of genetic structure and discreteness of wild trout populations identified here highlight that brook trout populations may be especially susceptible to the genetic effects of hatchery stocking if introgression occurs. Thus, future changes in environment, anthropogenic factors, or stocking practices could lead to changes in genetic introgression, and therefore, continued efforts focused on understanding the fate of hatchery trout in the wild and the ecological impacts of stocking on wild populations are needed to ensure proper management of this highly valued species. Overall, our study highlights how the genetic source of the hatchery stock can influence the likelihood to hatchery introgression, and further emphasizes the variable consequences of stocking and the need to assess the outcomes on a case‐specific basis.

## CONFLICT OF INTEREST

We have no conflict of interest to report.

## Data Availability

Microsatellite data and environmental/anthropogenic data are available from the Dryad Digital Repository: https://doi.org/10.5061/dryad.rv15dv44w
