# Supplementary material for: Multiple decades of stocking has resulted in limited hatchery introgression in wild brook trout (Salvelinus fontinalis) populations of Nova Scotia
Source: Evol Appl. 2020 Feb 20;13(5):1069–89. doi: 10.1111/eva.12923 (PMC7232767; doi:10.1111/eva.12923)
Supplement: Supplementary file 1 [file EVA-13-1069-s001.docx]

# SUPPLEMENTARY MATERIALS

**Table S1.** Brook trout (*Salvelinus fontinalis*) sampling sites (wild and hatchery) across Nova Scotia with location information and number of fish genotyped (N).

| **Code** | **River system** | **Tributary/Lake** | **Lat** | **Long** | **N** |
| --- | --- | --- | --- | --- | --- |
| Duf | St Mary’s Bay | Duffy Brook | 44.249 | -66.126 | 50 |
| Bou1 | St Mary’s Bay | Boudreau Brook site 1 | 44.266 | -66.114 | 29 |
| Bou2 | St Mary’s Bay | Boudreau Brook site 2 | 44.270 | -66.107 | 20 |
| FO | Annapolis River | Foster Brook | 44.835 | -65.361 | 44 |
| WA | Annapolis River | Walker Brook | 44.998 | -64.967 | 46 |
| Par | Annapolis River | Parker Brook | 45.050 | -64.815 | 47 |
| UM*^Ψ^ | Upper Medway | Multiple sites | 44.580 | -65.142 | 128 |
| RA | Cornwallis River | Rand Brook | 45.062 | -64.775 | 46 |
| Roc | Cornwallis River | Rochford Brook | 45.049 | -64.664 | 52 |
| BR | Cornwallis River | Brandywine Brook | 45.111 | -64.588 | 44 |
| Var | LaHave | Varner Brook Wild adult | 44.557 | -64.769 | 38 |
| VarYOY | LaHave | Varner Brook YOY | 44.557 | -64.769 | 12 |
| BU | LaHave | Butler Lake Brook | 44.727 | -64.660 | 68 |
| CO | LaHave | Cooks Brook | 44.412 | -64.553 | 48 |
| Far | Salmon River Truro | Farnham Brook | 45.385 | -63.244 | 48 |
| MC* | Musquodoboit River | McNutt Brook/Dollar Lake Brook | 45.043 | -63.164 | 47 |
| GE | Musquodoboit River | Gleason Brook | 45.161 | -62.910 | 46 |
| BI | East River Pictou | Big Brook | 45.414 | -62.738 | 47 |
| GL | East River Pictou | Glencoe Brook | 45.423 | -62.549 | 47 |
| Tho | East River Pictou | Thompson Brook | 45.404 | -62.451 | 46 |
| MO | Saint Mary’s River (East) | Moose River | 45.496 | -62.370 | 42 |
| Kel | Saint Mary’s River | Kelly Brook | 45.244 | -62.311 | 24 |
| GR | Saint Mary’s River (East) | Greens Brook | 45.423 | -62.207 | 47 |
| Cla | Saint Mary’s River | Clarks Creek | 45.264 | -62.196 | 20 |
| SA | River Denys | Sawmill Site | 45.861 | -61.247 | 55 |
| AL | River Denys | Alder Brook | 45.904 | -61.229 | 45 |
| RD*^Ψ^ | River Denys | Multiple sites | 45.872 | -61.073 | 50 |
| LakH^Φ^ | Margaree River | Lake O Law Brook | 46.332 | -61.000 | 31 |
| LakW | Margaree River | Lake O Law Brook | 46.332 | -61.000 | 55 |
| PO | Margaree River | Portree Brook | 46.397 | -60.972 | 48 |
| HA | Baddeck River | Harris Brook | 46.113 | -60.830 | 50 |
| Ang | Baddeck River | Angus Farquhars Brook | 46.133 | -60.800 | 48 |
| MI | Baddeck River | Mill Brook | 46.196 | -60.739 | 52 |
| FM | Hatchery | Fraser’s Mills Hatchery | 45.494 | -61.938 | 176 |
| MR | Hatchery | Margaree River Hatchery | 46.372 | -60.968 | 33 |
|  | | | | **Total** | **1729** |
| *Multiple sites sampled; ^Φ^ Evidence of fin erosion (possible hatchery fish);  ^Ψ^Average lat/long of multiple sites | | | |  |  |

**Table S2.** Microsatellite loci for brook trout (*Salvelinus fontinalis*). Locus_name codes include the multiplex (L, Salv-1, Salv-2, Sfon-6), the species that the original locus was designed for and the linkage group in the original species: SFO = *S. fontinalis*; Ssa-# = *Salmo salar* – linkage group (in *S. salar*), Salv-# and Sfon-# = *Salvelinus alpinus* – linkage group (in *S. alpinus*); di and tri refer to repeat type. Oligos sequences are 5’-3’.

| Locus_Name | Left_oligo | Right oligo | Accession | Ref for sequences |
| --- | --- | --- | --- | --- |
| L-SFOC113 | GGGGAGCCCAGACTATATTGA | TCATCATTGCCATCAGGGTA | AY168193 | King et al. (2012) |
| L-SFOC24 | AACACTGGAGCCGTTGAAGT | AGAGATGGGGTGATGCCTAA | AY168187 | King et al. (2012) |
| L-SFOC28 | CAGTTGAAGTGATTGGGTTAGC | CACACCACACACACAAAAGC | AY168188 | King et al. (2012) |
| L-SFOC88 | GGGAGAACCCAGTGTTTCTTT | CTGAACCCTGATTGTGAACG | AY168192 | King et al. (2012) |
| L-SFOD129 | GTGCAGGCACTAACTGGACA | CCAGGGAATCCTCATCTTCA | AY168195 | King et al. (2012) |
| L-Ssa-1.14 | TCGTATTTGTCAAGGATGTGCC | AGATGCCCATTGTATTGCCC | gi\|925168868\|ref\|NW_012332713.1\| | Lien et al. (2016) |
| L-Ssa-1.7 | AGAACACAACAGAACCAGGTAC | CTCGAACACACTTCCAACCC | gi\|925169069\|ref\|NW_012332512.1\| | Lien et al. (2016) |
| L-Ssa-10.3 | TGATGGGTCTTGGTGTAGGG | TCAACGGTGAAGCCCAGG | gi\|925165081\|ref\|NW_012336500.1\| | Lien et al. (2016) |
| L-Ssa-11.1 | AGAGCTCCGACACACATTCG | CATAGAGCTAGGCCGGTGC | gi\|925164508\|ref\|NW_012337073.1\| | Lien et al. (2016) |
| L-Ssa-12.11 | GTCTGAGGAAGCTGGCTCTG | TGTCCAGCAGCAACAACG | gi\|925164072\|ref\|NW_012337509.1\| | Lien et al. (2016) |
| L-Ssa-12.2 | ACTGGTAGGTCATTGTTCTGTG | CCTTATGTGTGTTCTCGGTGC | gi\|925164070\|ref\|NW_012337511.1\| | Lien et al. (2016) |
| L-Ssa-12.4 | TGCTTGCACCTAATAGTCCTAC | CTTGCGAGACATTGATTCAGC | gi\|925164081\|ref\|NW_012337500.1\| | Lien et al. (2016) |
| L-Ssa-13.6 | GCTGTTCCTCTGGCCTCAC | AGCACCTCAACACTGTACTATC | gi\|925163844\|ref\|NW_012337737.1\| | Lien et al. (2016) |
| L-Ssa-14.10 | GGGAACGTGTGGAAGATTCAC | AAGGTATGGAGGGTGATGCC | gi\|925163717\|ref\|NW_012337864.1\| | Lien et al. (2016) |
| L-Ssa-15.1 | TTTCTTTGTGTGTTGTGCCC | CAGCTGTGGTTCCTCTGGG | gi\|925163276\|ref\|NW_012338305.1\| | Lien et al. (2016) |
| L-Ssa-15.7 | GATGTGATGGCAGTGCTATG | CAGCAACAAGGTCAATCTCC | gi\|925163309\|ref\|NW_012338272.1\| | Lien et al. (2016) |
| L-Ssa-15.9 | ATACTACCTGTTCAGGCGGC | CCTGTGATGACTCTCCTCCC | gi\|925163457\|ref\|NW_012338124.1\| | Lien et al. (2016) |
| L-Ssa-16.2 | GTTTACGTCACCTGCAGCTG | GCAGTAGATGTTAAGCCCTCG | gi\|925163085\|ref\|NW_012338496.1\| | Lien et al. (2016) |
| L-Ssa-20.3 | GGAGGGAGTGTAGAGGCTTTC | CACACACCCATCTCTGCTAC | gi\|925161468\|ref\|NW_012340113.1\| | Lien et al. (2016) |
| L-Ssa-20.d16 | GGCAACGAGGTGAGAATGC | GTGCGTCTTACCTAGTTGCC | gi\|925161497\|ref\|NW_012340084.1\| | Lien et al. (2016) |
| L-Ssa-21.5 | CACTCCCTAACTCCATGGTC | TCATGGATGTCGTCACTGTG | gi\|925161233\|ref\|NW_012340348.1\| | Lien et al. (2016) |
| L-Ssa-23.9 | ACGGATACAGAGAGACGCAC | ACAGCGAGGAGGACAAAGTC | gi\|925161018\|ref\|NW_012340563.1\| | Lien et al. (2016) |
| L-Ssa-26.d06 | CATAATCACCTTGCATGACACC | CCTGCTGCACCGCTAAATAC | gi\|925160645\|ref\|NW_012340936.1\| | Lien et al. (2016) |
| L-Ssa-27.1 | TCCATGAGTACACGCCACTG | GTTCTCCACTACTCTACCCTGG | gi\|925160116\|ref\|NW_012341465.1\| | Lien et al. (2016) |
| L-Ssa-27.d07 | GATTTCACAAAGCAGCGCG | CAGCATGTTCTGTCGCGAG | gi\|925160121\|ref\|NW_012341460.1\| | Lien et al. (2016) |
| L-Ssa-27.d19 | GGAATACTGTCTCATTGCGCC | AGGCAATCAAAGGTTGTAGGTG | gi\|925160109\|ref\|NW_012341472.1\| | Lien et al. (2016) |
| L-Ssa-28.d08 | TCTGACCTACACACAACAATGG | GTGTGTGCTCGCTCAGAATG | gi\|925159981\|ref\|NW_012341600.1\| | Lien et al. (2016) |
| L-Ssa-29.2 | GGCACAGCACACCAGTTG | ACAGCGTTCCAAGATGTTCC | gi\|925159889\|ref\|NW_012341692.1\| | Lien et al. (2016) |
| L-Ssa-3.7 | GCACATTGAAGTTGGTTGCC | TTAACCAGAAGCTGCTCGTC | gi\|925168010\|ref\|NW_012333571.1\| | Lien et al. (2016) |
| L-Ssa-4.9 | AGAATCTCTAGCCCACACAAC | GCAGGGTTGAGATGTGAGC | gi\|925167363\|ref\|NW_012334218.1\| | Lien et al. (2016) |
| L-Ssa-4.d56 | CTGCTGGTAAATGGGCGTTG | CCTGTCCTGGTTAACAAAGGC | gi\|925167308\|ref\|NW_012334273.1\| | Lien et al. (2016) |
| L-Ssa-6.8 | TGAGGCCGATGTCACCTG | AGTACCTGAACTCGACGGC | gi\|925166209\|ref\|NW_012335372.1\| | Lien et al. (2016) |
| L-Ssa-9.12 | CTGACAGGTGGAGTGGGAC | TCCCGCATGTACCTGCAG | gi\|925165260\|ref\|NW_012336321.1\| | Lien et al. (2016) |
| Salv-1_Di-03-1639 | CGCCACAGGTTGCTAAGATTG | GCGTGTATTCAGAAGCAGCAC | CM009420.1 | Christensen et al. (2018) |
| Salv-1_Di-05-1487 | CTTCATGCTCATCACTGGCC | CATGCACCAAACACCACTCC | CM009432.1 | Christensen et al. (2018) |
| Salv-1_Di-08-1675 | ACACAGCTTCAAGTGTAGAGTG | TGGGTGACAAGAGACCGTG | CM009436.1 | Christensen et al. (2018) |
| Salv-1_Di-16-475 | AGAGTGGAGGCTGTGTATGC | TCACAAACACGCTCGCTTG | CM009406.1 | Christensen et al. (2018) |
| Salv-1_Di-19-418 | GAACGCCAAGCTAGAGAGGG | CATCAACTCCAGCGCCATTAG | CM009409.1 | Christensen et al. (2018) |
| Salv-1_Di-20-1207 | CCTTCACTATGGCTGGGCAC | GGAGCGACATATTTCAACTCAAG | CM009411.1 | Christensen et al. (2018) |
| Salv-1_Di-30-1164 | GCTTGATTCGCTGTTGTCTC | AAATCCATGTTTACAGTCACGC | CM009421.1 | Christensen et al. (2018) |
| Salv-1_Di-30-1186 | ATTCAGCCAACACAGAGACAG | GTGGAATCAAGGCCCTTTCTC | CM009421.1 | Christensen et al. (2018) |
| Salv-1_Di-31-422 | CACCATTAGCCAGCGGTG | ACCTTGTTCTGCTCCATCAC | CM009422.1 | Christensen et al. (2018) |
| Salv-1_Di-31-502 | GCCTATGACACGGTAGAAATGC | ACATTATGGCCGCTGTCAATC | CM009422.1 | Christensen et al. (2018) |
| Salv-1_Di-32-188 | AGGAGTTTGTCTGGGCACAC | GACAGACAGTAACCATCCATAGC | CM009423.1 | Christensen et al. (2018) |
| Salv-1_Di-33-1038 | TCGGTTTCAGTTGACTCCCAC | CCTGCCTCCTGTTCTTTCAAG | CM009424.1 | Christensen et al. (2018) |
| Salv-1_Di-36-244 | GCTGTCTGTCACACCTCAAAC | GGCGTGTTAGGTCTATTTCTCC | CM009427.1 | Christensen et al. (2018) |
| Salv-1_Di-36-765 | AGGAATATCATCACAGTGCAGC | AGTCTGTCATTTCCTGCTCTTG | CM009427.1 | Christensen et al. (2018) |
| Salv-1_tri-01-26 | ATGGCTAACTTGCGCAATGTC | ACTTTATCGACCACTGCAACG | CM009399.1 | Christensen et al. (2018) |
| Salv-1_tri-11-1 | GTGGCGGCATGTACTTCTAAC | GATACAGGGTTTGACGGACTC | CM009401.1 | Christensen et al. (2018) |
| Salv-1_tri-11-16 | CCGTTGGCAAGGTGATTAAC | ATCAAGGTCAATGCAACGGTC | CM009401.1 | Christensen et al. (2018) |
| Salv-1_Tri-12-2 | ATGAGAGTGCAACCCAACAAC | AACACAGACTTCCTCCCAGAC | CM009402.1 | Christensen et al. (2018) |
| Salv-1_tri-13-14 | AGTTCCGCTCCTCTCCATTC | AGGAGGGAGGATTGGAGGG | CM009403.1 | Christensen et al. (2018) |
| Salv-1_tri-14-6 | CTAATGTGGCTGAGTCAATTGC | TCGTCGTTCATGTCCTTATCATC | CM009404.1 | Christensen et al. (2018) |
| Salv-1_tri-16-9 | CCTGCAGCAGTAAGAGAGGAC | GTAGATCATGGCCCAGGGTG | CM009406.1 | Christensen et al. (2018) |
| Salv-1_tri-17-17 | GGTTCTCCAGGGTCTGCAG | GAGGAGTACGCCCACACAG | CM009407.1 | Christensen et al. (2018) |
| Salv-1_tri-18-13 | ATCCCAGCGGTTGTTGAC | CATCTGTAGCCGCCGATTAC | CM009408.1 | Christensen et al. (2018) |
| Salv-2_tri-02-5 | AGACTACTTCAAGCACCAGAC | AGAGAAAGGGAGTGAGCTTAAC | CM009410.1 | Christensen et al. (2018) |
| Salv-2_tri-03-20 | GTGATTCACGAACGAGGGATC | TCTCACCCAGCTCTATTCTCC | CM009420.1 | Christensen et al. (2018) |
| Salv-2_tri-03-22 | GAGTCCCTCCAGTCCATTTATG | ATTACCACCACCGACCCTTC | CM009420.1 | Christensen et al. (2018) |
| Salv-2_tri-05-4 | GCAAATGACTTCCCTCTCTGC | CTTCTATCTCTCTCGGCCGG | CM009432.1 | Christensen et al. (2018) |
| Salv-2_tri-07-12 | CGACATGAAGGAAGCTGCAC | CTTCAGTCTGTTCAGTGGAGC | CM009435.1 | Christensen et al. (2018) |
| Salv-2_tri-20-21 | GAGAGCGAGGGATTGGTATGG | GACAACCAGCCACCTCCC | CM009411.1 | Christensen et al. (2018) |
| Salv-2_tri-20-30 | CTGGACCGGCATAATACAGAAG | TTCACGTTTGTTCCTTTCCCG | CM009411.1 | Christensen et al. (2018) |
| Salv-2_tri-20-5 | TGGCCACATATGCTCCCG | CTTGTGATCGCCAACCACC | CM009411.1 | Christensen et al. (2018) |
| Salv-2_tri-22-20 | CCATCATTGAGAGCTAGCTGTG | GAGCTCCCAAGTCTAGTGTTTC | NC_036862.1 | Christensen et al. (2018) |
| Salv-2_tri-23-0 | GCCGATAAACACCACCGAAG | TCCTGGCTTGATTGAATACTTGC | CM009414.1 | Christensen et al. (2018) |
| Salv-2_tri-23-5 | GCGCTTCTGTTCTATCCTCAC | AGCTCGACTTGCAGGACTC | CM009414.1 | Christensen et al. (2018) |
| Salv-2_tri-24-1 | GTGACCGGCTTGTTCCATTC | TGCCTCGTACTCACTACAGAC | CM009415.1 | Christensen et al. (2018) |
| Salv-2_tri-25-5 | GGAAGGCCAGTACAATATTGAGG | TTGGTTACTGCGGTTATGGTC | CM009416.1 | Christensen et al. (2018) |
| Salv-2_tri-28-0 | CTCGGTGACTTTCCCAGATTC | TCCAGAGTCTACAACAATGACC | CM009419.1 | Christensen et al. (2018) |
| Salv-2_tri-28-9 | CGGCCAATAGTCCTGTTCATC | ACTTTCCTAAACAAGCACCATTG | CM009419.1 | Christensen et al. (2018) |
| Salv-2_tri-30-1 | GATGACACTGCAAACTGGGAG | AAAGCTCCAAGTGCCTCCTTC | CM009421.1 | Christensen et al. (2018) |
| Salv-2_tri-31-5 | AACAGCCTGAGTGTTGTTGTC | ATAGTCCTCCTCACAGACACG | CM009422.1 | Christensen et al. (2018) |
| Salv-2_tri-31-9 | AAAGCGCTATATAGGTCCCATG | CACTCCAAGCTGACAATCATATG | CM009422.1 | Christensen et al. (2018) |
| Salv-2_tri-4q2-12 | AGAACAGGTATAAGCAACCACC | CAGAATCACTCATAAGGACCAGG | CM009431.1 | Christensen et al. (2018) |
| Sfon-6_Di-01-402 | ATGTGCATTTGTGTACCTCCG | AGTTCACTCCCGTCCACAC | CM009399.1 | Christensen et al. (2018) |
| Sfon-6_Di-03-296 | AGCTGTGTACTATCAGGTCTCC | TGTCATTCTGTTCCATTCCAGG | CM009420.1 | Christensen et al. (2018) |
| Sfon-6_Di-07-134 | TGAGAACAAACACACAGCTGC | CTCCGCTGTTTGAGATGTGC | CM009435.1 | Christensen et al. (2018) |
| Sfon-6_Di-18-1313 | CACACAGGGAAACAGTCTGAG | GTTACATCCAAGGAAACGGCG | CM009408.1 | Christensen et al. (2018) |
| Sfon-6_Di-18-683 | TCATGCGTCACTGTCACAAAC | GAGAAGGAATGGATGGATCACC | CM009408.1 | Christensen et al. (2018) |
| Sfon-6_Di-20-379 | AGATAGATGGGCCCTACTGATTG | AGAGCTGCAACCTCACCAAG | CM009411.1 | Christensen et al. (2018) |
| Sfon-6_Di-24-298 | AATGCTCTAACGGATGCTGTG | GTAAGAGACTTGGGCTTGTGTG | CM009415.1 | Christensen et al. (2018) |
| Sfon-6_Di-28-785 | ACATCTGATCTCTGTGTCTGGG | ATAGACAGCAGGCCATCAGAC | CM009419.1 | Christensen et al. (2018) |
| Sfon-6_Di-30-152 | GTTGTGACAAGGAAGGATCTGAG | TGAAACTACCACATATGCACGC | CM009421.1 | Christensen et al. (2018) |
| Sfon-6_Di-36-592 | TGGTTTCACTCACTCCCAAG | ACTTGTGGTCATAGCCCACTG | CM009427.1 | Christensen et al. (2018) |
| Sfon-6_Di-37-567 | GTGTATGCAATCTTGTGTGATGC | TACACTGCTCTGAAAGGGAGG | CM009428.1 | Christensen et al. (2018) |
| Sfon-6_Di-4q-1577 | CTCGCATATGTTTATTGTGGTGC | ACACTGAAACCGCTTACCTAG | CM009430.1 | Christensen et al. (2018) |
| Sfon-6_Di-6.1-1018 | CTGCTCATTACTGGTGCTCTC | TAGAATGATCGAATGTGCGCG | CM009433.1 | Christensen et al. (2018) |
| Sfon-6_tri-01-16 | GACCTGCCTGCAATGTAGC | GATGTTCTACTTCCCAGCTGC | CM009399.1 | Christensen et al. (2018) |
| Sfon-6_tri-01-18 | GGAGGCCCAAACTTGTATATGG | CCACCAATATGCCCTTGAAATAG | CM009399.1 | Christensen et al. (2018) |
| Sfon-6_tri-10-2 | AACAAGACACCACCATCCTC | TCCATTCTATTTAGTCTGCGGTC | CM009400.1 | Christensen et al. (2018) |
| Sfon-6_tri-16-3 | AACATGGTCACTGGAGGACTC | CTCAGGAAGTTTAGCCATTCATC | CM009406.1 | Christensen et al. (2018) |
| Sfon-6_tri-20-16 | CAAGACTTGAGAGACCAGTGG | TTGTGTGACAAAGTGACAGCC | CM009411.1 | Christensen et al. (2018) |
| Sfon-6_tri-20-20 | CATGTTGCTATGGAGGAGGG | CCTCTGCCTTTGAATTTGCTC | CM009411.1 | Christensen et al. (2018) |
| Sfon-6_tri-26-12 | CTTATGGGCATGACACCTCTG | TGGTATTGAGCTCCACTGTCC | CM009417.1 | Christensen et al. (2018) |
| Sfon-6_tri-32-0 | TAATGGACATCTGAGCCTCCG | GGCTGGCTTCATATGTTCTGC | CM009423.1 | Christensen et al. (2018) |
| Sfon-6_tri-33-10 | TTGTTCTGCTCGTCTTTCTGC | AAAGGCAGTCAAGGAGGAGAG | CM009424.1 | Christensen et al. (2018) |
| Sfon-6_tri-4p-3 | TGCCAACTGTACACATGATTCC | ATGAAGTGGTGATGTGGCATG | CM009429.1 | Christensen et al. (2018) |
| Sfon-6_tri-4q-0 | GTTCTGAGTTTAGGACACGCC | TGTGGTAGAACTGTGTGGATATG | CM009430.1 | Christensen et al. (2018) |
| Sfon-6_tri-6.1-3 | GCATCATCCCAGGTGATTGTC | GTTGGTGAATAGTGAGGGTCAG | CM009433.1 | Christensen et al. (2018) |

**Table S3.** Pairwise *F*_ST_ between all brook trout (*Salvelinus fontinalis*) sampling sites in Nova Scotia rivers. All comparisons were significant (alpha level=0.0014) with the exception of shaded cells (8 comparisons).

| **Pop** | **Duf** | **Bou1** | **Bou2** | **FO** | **WA** | **Par** | **UM** | **RA** | **Roc** | **BR** | **Var** | **VarYOY** | **BU** | **CO** | **Far** | **MC** |
| --- | --- | --- | --- | --- | --- | --- | --- | --- | --- | --- | --- | --- | --- | --- | --- | --- |
| **Duf** | - |  |  |  |  |  |  |  |  |  |  |  |  |  |  |  |
| **Bou1** | 0.178 | - |  |  |  |  |  |  |  |  |  |  |  |  |  |  |
| **Bou2** | 0.201 | 0.012 | - |  |  |  |  |  |  |  |  |  |  |  |  |  |
| **FO** | 0.200 | 0.141 | 0.169 | - |  |  |  |  |  |  |  |  |  |  |  |  |
| **WA** | 0.198 | 0.147 | 0.169 | 0.021 | - |  |  |  |  |  |  |  |  |  |  |  |
| **Par** | 0.203 | 0.147 | 0.175 | 0.037 | 0.023 | - |  |  |  |  |  |  |  |  |  |  |
| **UM** | 0.212 | 0.154 | 0.172 | 0.111 | 0.102 | 0.102 | - |  |  |  |  |  |  |  |  |  |
| **RA** | 0.226 | 0.161 | 0.182 | 0.089 | 0.079 | 0.088 | 0.135 | - |  |  |  |  |  |  |  |  |
| **Roc** | 0.217 | 0.146 | 0.166 | 0.062 | 0.060 | 0.071 | 0.123 | 0.029 | - |  |  |  |  |  |  |  |
| **BR** | 0.239 | 0.179 | 0.204 | 0.077 | 0.067 | 0.082 | 0.125 | 0.039 | 0.023 | - |  |  |  |  |  |  |
| **Var** | 0.231 | 0.160 | 0.177 | 0.116 | 0.107 | 0.120 | 0.164 | 0.158 | 0.146 | 0.161 | - |  |  |  |  |  |
| **VarYOY** | 0.235 | 0.164 | 0.188 | 0.138 | 0.121 | 0.142 | 0.185 | 0.171 | 0.165 | 0.176 | 0.038 | - |  |  |  |  |
| **BU** | 0.224 | 0.145 | 0.175 | 0.094 | 0.092 | 0.102 | 0.117 | 0.138 | 0.118 | 0.122 | 0.148 | 0.161 | - |  |  |  |
| **CO** | 0.201 | 0.112 | 0.141 | 0.067 | 0.066 | 0.070 | 0.083 | 0.108 | 0.096 | 0.106 | 0.086 | 0.105 | 0.089 | - |  |  |
| **Far** | 0.235 | 0.179 | 0.191 | 0.080 | 0.073 | 0.091 | 0.134 | 0.082 | 0.075 | 0.067 | 0.155 | 0.174 | 0.139 | 0.100 | - |  |
| **MC** | 0.219 | 0.187 | 0.208 | 0.135 | 0.111 | 0.115 | 0.101 | 0.124 | 0.122 | 0.126 | 0.164 | 0.183 | 0.132 | 0.092 | 0.122 | - |
| **GE** | 0.224 | 0.175 | 0.198 | 0.115 | 0.094 | 0.089 | 0.071 | 0.102 | 0.101 | 0.099 | 0.159 | 0.180 | 0.110 | 0.062 | 0.095 | 0.011 |
| **BI** | 0.206 | 0.169 | 0.190 | 0.131 | 0.137 | 0.135 | 0.141 | 0.152 | 0.147 | 0.164 | 0.193 | 0.201 | 0.142 | 0.134 | 0.142 | 0.123 |
| **GL** | 0.267 | 0.248 | 0.269 | 0.230 | 0.222 | 0.234 | 0.212 | 0.206 | 0.208 | 0.219 | 0.287 | 0.304 | 0.233 | 0.221 | 0.224 | 0.178 |
| **Tho** | 0.210 | 0.163 | 0.186 | 0.131 | 0.128 | 0.132 | 0.125 | 0.135 | 0.129 | 0.147 | 0.194 | 0.206 | 0.138 | 0.122 | 0.132 | 0.098 |
| **MO** | 0.194 | 0.161 | 0.185 | 0.111 | 0.103 | 0.093 | 0.066 | 0.114 | 0.117 | 0.120 | 0.175 | 0.201 | 0.122 | 0.093 | 0.121 | 0.095 |
| **Kel** | 0.266 | 0.205 | 0.233 | 0.165 | 0.161 | 0.159 | 0.113 | 0.176 | 0.180 | 0.183 | 0.204 | 0.233 | 0.168 | 0.119 | 0.183 | 0.119 |
| **GR** | 0.333 | 0.307 | 0.345 | 0.277 | 0.274 | 0.274 | 0.239 | 0.304 | 0.301 | 0.324 | 0.360 | 0.406 | 0.286 | 0.269 | 0.280 | 0.248 |
| **Cla** | 0.216 | 0.147 | 0.171 | 0.103 | 0.094 | 0.097 | 0.064 | 0.105 | 0.111 | 0.110 | 0.166 | 0.172 | 0.110 | 0.074 | 0.104 | 0.047 |
| **SA** | 0.211 | 0.176 | 0.203 | 0.134 | 0.123 | 0.130 | 0.120 | 0.122 | 0.123 | 0.130 | 0.199 | 0.213 | 0.139 | 0.123 | 0.139 | 0.103 |
| **AL** | 0.202 | 0.166 | 0.197 | 0.129 | 0.119 | 0.130 | 0.119 | 0.127 | 0.125 | 0.132 | 0.193 | 0.202 | 0.137 | 0.122 | 0.142 | 0.101 |
| **RD** | 0.207 | 0.172 | 0.201 | 0.132 | 0.125 | 0.133 | 0.117 | 0.123 | 0.126 | 0.131 | 0.195 | 0.206 | 0.135 | 0.118 | 0.141 | 0.097 |
| **LakH** | 0.209 | 0.175 | 0.198 | 0.163 | 0.161 | 0.164 | 0.157 | 0.153 | 0.153 | 0.161 | 0.236 | 0.231 | 0.151 | 0.162 | 0.173 | 0.130 |
| **LakW** | 0.212 | 0.163 | 0.184 | 0.145 | 0.142 | 0.147 | 0.142 | 0.138 | 0.141 | 0.148 | 0.215 | 0.213 | 0.146 | 0.145 | 0.145 | 0.115 |
| **PO** | 0.203 | 0.154 | 0.175 | 0.132 | 0.133 | 0.137 | 0.133 | 0.140 | 0.136 | 0.140 | 0.198 | 0.196 | 0.121 | 0.134 | 0.137 | 0.110 |
| **HA** | 0.179 | 0.137 | 0.164 | 0.079 | 0.070 | 0.087 | 0.076 | 0.076 | 0.079 | 0.087 | 0.166 | 0.179 | 0.098 | 0.082 | 0.098 | 0.062 |
| **Ang** | 0.184 | 0.154 | 0.181 | 0.116 | 0.108 | 0.124 | 0.109 | 0.115 | 0.118 | 0.123 | 0.183 | 0.196 | 0.115 | 0.110 | 0.129 | 0.088 |
| **MI** | 0.201 | 0.167 | 0.195 | 0.128 | 0.121 | 0.130 | 0.106 | 0.117 | 0.121 | 0.127 | 0.196 | 0.210 | 0.133 | 0.122 | 0.133 | 0.096 |
| **MR** | 0.158 | 0.125 | 0.143 | 0.123 | 0.117 | 0.117 | 0.116 | 0.116 | 0.112 | 0.122 | 0.176 | 0.175 | 0.107 | 0.115 | 0.127 | 0.119 |
| **FraserMills** | 0.160 | 0.131 | 0.144 | 0.175 | 0.157 | 0.157 | 0.181 | 0.161 | 0.153 | 0.170 | 0.180 | 0.185 | 0.144 | 0.158 | 0.187 | 0.197 |
| *Continued* |  |  |  |  |  |  |  |  |  |  |  |  |  |  |  |  |

| **Pop** | **GE** | **BI** | **GL** | **Tho** | **MO** | **Kel** | **GR** | **Cla** | **SA** | **AL** | **RD** | **LakH** | **LakW** | **PO** | **HA** | **Ang** |
| --- | --- | --- | --- | --- | --- | --- | --- | --- | --- | --- | --- | --- | --- | --- | --- | --- |
| **Duf** |  |  |  |  |  |  |  |  |  |  |  |  |  |  |  |  |
| **Bou1** |  |  |  |  |  |  |  |  |  |  |  |  |  |  |  |  |
| **Bou2** |  |  |  |  |  |  |  |  |  |  |  |  |  |  |  |  |
| **FO** |  |  |  |  |  |  |  |  |  |  |  |  |  |  |  |  |
| **WA** |  |  |  |  |  |  |  |  |  |  |  |  |  |  |  |  |
| **Par** |  |  |  |  |  |  |  |  |  |  |  |  |  |  |  |  |
| **UM** |  |  |  |  |  |  |  |  |  |  |  |  |  |  |  |  |
| **RA** |  |  |  |  |  |  |  |  |  |  |  |  |  |  |  |  |
| **Roc** |  |  |  |  |  |  |  |  |  |  |  |  |  |  |  |  |
| **BR** |  |  |  |  |  |  |  |  |  |  |  |  |  |  |  |  |
| **Var** |  |  |  |  |  |  |  |  |  |  |  |  |  |  |  |  |
| **VarYOY** |  |  |  |  |  |  |  |  |  |  |  |  |  |  |  |  |
| **BU** |  |  |  |  |  |  |  |  |  |  |  |  |  |  |  |  |
| **CO** |  |  |  |  |  |  |  |  |  |  |  |  |  |  |  |  |
| **Far** |  |  |  |  |  |  |  |  |  |  |  |  |  |  |  |  |
| **MC** |  |  |  |  |  |  |  |  |  |  |  |  |  |  |  |  |
| **GE** | - |  |  |  |  |  |  |  |  |  |  |  |  |  |  |  |
| **BI** | 0.103 | - |  |  |  |  |  |  |  |  |  |  |  |  |  |  |
| **GL** | 0.181 | 0.157 | - |  |  |  |  |  |  |  |  |  |  |  |  |  |
| **Tho** | 0.072 | 0.021 | 0.157 | - |  |  |  |  |  |  |  |  |  |  |  |  |
| **MO** | 0.072 | 0.081 | 0.171 | 0.072 | - |  |  |  |  |  |  |  |  |  |  |  |
| **Kel** | 0.094 | 0.104 | 0.217 | 0.104 | 0.097 | - |  |  |  |  |  |  |  |  |  |  |
| **GR** | 0.249 | 0.252 | 0.365 | 0.251 | 0.244 | 0.319 | - |  |  |  |  |  |  |  |  |  |
| **Cla** | 0.015 | 0.075 | 0.165 | 0.069 | 0.028 | 0.052 | 0.235 | - |  |  |  |  |  |  |  |  |
| **SA** | 0.082 | 0.082 | 0.167 | 0.061 | 0.070 | 0.127 | 0.276 | 0.081 | - |  |  |  |  |  |  |  |
| **AL** | 0.084 | 0.078 | 0.166 | 0.061 | 0.074 | 0.136 | 0.276 | 0.083 | 0.008 | - |  |  |  |  |  |  |
| **RD** | 0.078 | 0.074 | 0.163 | 0.052 | 0.070 | 0.126 | 0.275 | 0.080 | 0.000 | 0.006 | - |  |  |  |  |  |
| **LakH** | 0.112 | 0.090 | 0.180 | 0.087 | 0.104 | 0.163 | 0.289 | 0.111 | 0.105 | 0.095 | 0.095 | - |  |  |  |  |
| **LakW** | 0.099 | 0.079 | 0.176 | 0.075 | 0.100 | 0.136 | 0.271 | 0.098 | 0.096 | 0.080 | 0.085 | 0.019 | - |  |  |  |
| **PO** | 0.095 | 0.065 | 0.167 | 0.062 | 0.090 | 0.121 | 0.265 | 0.088 | 0.084 | 0.068 | 0.074 | 0.020 | 0.005 | - |  |  |
| **HA** | 0.045 | 0.034 | 0.129 | 0.019 | 0.033 | 0.095 | 0.245 | 0.043 | -0.007 | -0.008 | -0.010 | 0.047 | 0.034 | 0.022 | - |  |
| **Ang** | 0.066 | 0.065 | 0.141 | 0.045 | 0.066 | 0.113 | 0.252 | 0.072 | 0.022 | 0.021 | 0.017 | 0.076 | 0.067 | 0.056 | -0.028 | - |
| **MI** | 0.078 | 0.064 | 0.153 | 0.042 | 0.068 | 0.115 | 0.263 | 0.080 | 0.023 | 0.024 | 0.018 | 0.081 | 0.067 | 0.059 | -0.005 | 0.007 |
| **MR** | 0.107 | 0.071 | 0.153 | 0.060 | 0.090 | 0.121 | 0.259 | 0.073 | 0.078 | 0.064 | 0.068 | 0.007 | 0.011 | 0.009 | 0.009 | 0.044 |
| **FraserMills** | 0.196 | 0.185 | 0.237 | 0.180 | 0.175 | 0.230 | 0.295 | 0.167 | 0.179 | 0.169 | 0.174 | 0.158 | 0.162 | 0.148 | 0.127 | 0.161 |
| *Continued* |  |  |  |  |  |  |  |  |  |  |  |  |  |  |  |  |

| **Pop** | **MI** | **MR** | **FraserMills** |
| --- | --- | --- | --- |
| **Duf** |  |  |  |
| **Bou1** |  |  |  |
| **Bou2** |  |  |  |
| **FO** |  |  |  |
| **WA** |  |  |  |
| **Par** |  |  |  |
| **UM** |  |  |  |
| **RA** |  |  |  |
| **Roc** |  |  |  |
| **BR** |  |  |  |
| **Var** |  |  |  |
| **VarYOY** |  |  |  |
| **BU** |  |  |  |
| **CO** |  |  |  |
| **Far** |  |  |  |
| **MC** |  |  |  |
| **GE** |  |  |  |
| **BI** |  |  |  |
| **GL** |  |  |  |
| **Tho** |  |  |  |
| **MO** |  |  |  |
| **Kel** |  |  |  |
| **GR** |  |  |  |
| **Cla** |  |  |  |
| **SA** |  |  |  |
| **AL** |  |  |  |
| **RD** |  |  |  |
| **LakH** |  |  |  |
| **LakW** |  |  |  |
| **PO** |  |  |  |
| **HA** |  |  |  |
| **Ang** |  |  |  |
| **MI** | - |  |  |
| **MR** | 0.049 | - |  |
| **FraserMills** | 0.171 | 0.109 | - |

**Table S4.** Pairwise genetic divergence (*F*_ST_; lower triangle) and associated significance (*p*-values; upper triangle) for brook trout (*Salvelinus fontinalis*) hatchery strains at Fraser’s Mills hatchery. Significant genetic divergence between strains is indicated by bold text and an asterisk (*) and alpha level was adjusted for multiple comparisons (0.05/5=0.01).

|  | **Flat Lake strain** | **Fraser’s Mills domestic strain** | **Pictou Pond Derby** | **Timber Lake strain** | **Sea trout strain** |
| --- | --- | --- | --- | --- | --- |
| **Flat Lake strain (n=38)** | - | 0.000 | 0.999 | 0.016 | 0.000 |
| **Fraser’s Mills domestic strain 1 (n=49)** | **0.038*** | - | 0.193 | 0.000 | 0.000 |
| **Pictou Pond Derby (n=19)** | -0.038 | 0.004 | - | 0.771 | 0.995 |
| **Timber Lake strain (n=32)** | 0.007 | **0.045*** | -0.003 | - | 0.000 |
| **Sea trout strain (n=38)** | **0.026*** | **0.056*** | -0.009 | **0.040*** | - |

**Table S5.** Correlation coefficient (*r*) between all 19 bioclimatic variables (scaled) for brook trout (*Salvelinus fontinalis*) sampling sites.

|  | **BIO1** | **BIO2** | **BIO3** | **BIO4** | **BIO5** | **BIO6** | **BIO7** | **BIO8** | **BIO9** | **BIO10** | **BIO11** | **BIO12** | **BIO13** | **BIO14** | **BIO15** | **BIO16** | **BIO17** | **BIO18** | **BIO19** |
| --- | --- | --- | --- | --- | --- | --- | --- | --- | --- | --- | --- | --- | --- | --- | --- | --- | --- | --- | --- |
| **BIO1** |  | 0.13 | 0.50 | -0.25 | 0.36 | 0.61 | -0.19 | -0.31 | 0.23 | 0.54 | 0.73 | -0.67 | -0.64 | -0.46 | -0.54 | -0.72 | -0.52 | -0.80 | -0.34 |
| **BIO2** | 0.13 |  | 0.54 | 0.71 | 0.82 | -0.60 | 0.81 | -0.22 | 0.06 | 0.67 | -0.44 | -0.18 | -0.39 | 0.12 | -0.52 | -0.33 | 0.06 | -0.19 | -0.08 |
| **BIO3** | 0.50 | 0.54 |  | -0.19 | 0.20 | 0.25 | -0.06 | -0.27 | 0.32 | 0.15 | 0.37 | -0.38 | -0.44 | -0.19 | -0.48 | -0.49 | -0.24 | -0.58 | 0.02 |
| **BIO4** | -0.25 | 0.71 | -0.19 |  | 0.78 | -0.89 | 0.97 | 0.03 | -0.12 | 0.67 | -0.84 | 0.05 | -0.16 | 0.32 | -0.31 | -0.05 | 0.29 | 0.19 | -0.14 |
| **BIO5** | 0.36 | 0.82 | 0.20 | 0.78 |  | -0.49 | 0.83 | -0.23 | 0.02 | 0.96 | -0.33 | -0.24 | -0.44 | 0.05 | -0.53 | -0.37 | -0.01 | -0.22 | -0.23 |
| **BIO6** | 0.61 | -0.60 | 0.25 | -0.89 | -0.49 |  | -0.89 | -0.08 | 0.25 | -0.31 | 0.96 | -0.28 | -0.09 | -0.36 | 0.01 | -0.22 | -0.36 | -0.47 | 0.00 |
| **BIO7** | -0.19 | 0.81 | -0.06 | 0.97 | 0.83 | -0.89 |  | -0.07 | -0.15 | 0.69 | -0.79 | 0.05 | -0.17 | 0.26 | -0.28 | -0.05 | 0.23 | 0.18 | -0.12 |
| **BIO8** | -0.31 | -0.22 | -0.27 | 0.03 | -0.23 | -0.08 | -0.07 |  | -0.04 | -0.24 | -0.17 | 0.16 | 0.17 | 0.38 | 0.00 | 0.16 | 0.39 | 0.26 | -0.22 |
| **BIO9** | 0.23 | 0.06 | 0.32 | -0.12 | 0.02 | 0.25 | -0.15 | -0.04 |  | 0.04 | 0.21 | 0.10 | 0.06 | 0.29 | -0.22 | -0.01 | 0.24 | -0.18 | 0.35 |
| **BIO10** | 0.54 | 0.67 | 0.15 | 0.67 | 0.96 | -0.31 | 0.69 | -0.24 | 0.04 |  | -0.16 | -0.40 | -0.56 | -0.06 | -0.61 | -0.52 | -0.13 | -0.38 | -0.34 |
| **BIO11** | 0.73 | -0.44 | 0.37 | -0.84 | -0.33 | 0.96 | -0.79 | -0.17 | 0.21 | -0.16 |  | -0.36 | -0.18 | -0.45 | -0.04 | -0.31 | -0.46 | -0.53 | -0.08 |
| **BIO12** | -0.67 | -0.18 | -0.38 | 0.05 | -0.24 | -0.28 | 0.05 | 0.16 | 0.10 | -0.40 | -0.36 |  | 0.96 | 0.70 | 0.63 | 0.97 | 0.78 | 0.89 | 0.78 |
| **BIO13** | -0.64 | -0.39 | -0.44 | -0.16 | -0.44 | -0.09 | -0.17 | 0.17 | 0.06 | -0.56 | -0.18 | 0.96 |  | 0.54 | 0.79 | 0.99 | 0.63 | 0.85 | 0.74 |
| **BIO14** | -0.46 | 0.12 | -0.19 | 0.32 | 0.05 | -0.36 | 0.26 | 0.38 | 0.29 | -0.06 | -0.45 | 0.70 | 0.54 |  | -0.05 | 0.55 | 0.96 | 0.62 | 0.48 |
| **BIO15** | -0.54 | -0.52 | -0.48 | -0.31 | -0.53 | 0.01 | -0.28 | 0.00 | -0.22 | -0.61 | -0.04 | 0.63 | 0.79 | -0.05 |  | 0.79 | 0.06 | 0.63 | 0.44 |
| **BIO16** | -0.72 | -0.33 | -0.49 | -0.05 | -0.37 | -0.22 | -0.05 | 0.16 | -0.01 | -0.52 | -0.31 | 0.97 | 0.99 | 0.55 | 0.79 |  | 0.65 | 0.90 | 0.71 |
| **BIO17** | -0.52 | 0.06 | -0.24 | 0.29 | -0.01 | -0.36 | 0.23 | 0.39 | 0.24 | -0.13 | -0.46 | 0.78 | 0.63 | 0.96 | 0.06 | 0.65 |  | 0.71 | 0.56 |
| **BIO18** | -0.80 | -0.19 | -0.58 | 0.19 | -0.22 | -0.47 | 0.18 | 0.26 | -0.18 | -0.38 | -0.53 | 0.89 | 0.85 | 0.62 | 0.63 | 0.90 | 0.71 |  | 0.47 |
| **BIO19** | -0.34 | -0.08 | 0.02 | -0.14 | -0.23 | 0.00 | -0.12 | -0.22 | 0.35 | -0.34 | -0.08 | 0.78 | 0.74 | 0.48 | 0.44 | 0.71 | 0.56 | 0.47 |  |

**Table S6.** Correlation coefficient (*r*) between all 8 stocking variables (scaled) for brook trout (*Salvelinus fontinalis*) sampling sites.

|  | **Propagule pressure** | **Total fish stocked** | **Mean number stocked** | **Mean weight** | **Proportion adult** | **Spring stocking** | **Fall stocking** | **Years since mean stocking** |
| --- | --- | --- | --- | --- | --- | --- | --- | --- |
| **Propagule pressure** |  | 0.60 | 0.67 | 0.55 | 0.08 | 0.57 | 0.59 | -0.67 |
| **Total fish stocked** | 0.60 |  | 0.78 | 0.34 | -0.20 | 0.97 | 0.96 | -0.69 |
| **Mean number stocked** | 0.67 | 0.78 |  | 0.53 | 0.05 | 0.73 | 0.77 | -0.90 |
| **Mean weight** | 0.55 | 0.34 | 0.53 |  | 0.39 | 0.32 | 0.34 | -0.70 |
| **Proportion adult** | 0.08 | -0.20 | 0.05 | 0.39 |  | -0.15 | -0.23 | -0.27 |
| **Spring stocking** | 0.57 | 0.97 | 0.73 | 0.32 | -0.15 |  | 0.85 | -0.65 |
| **Fall stocking** | 0.59 | 0.96 | 0.77 | 0.34 | -0.23 | 0.85 |  | -0.67 |
| **Years since mean stocking** | -0.67 | -0.69 | -0.90 | -0.70 | -0.27 | -0.65 | -0.67 |  |

**Figure S1.** Size distribution of small (≤ 500 grams) and large (>500 grams) brook trout (*Salvelinus fontinalis*) that were stocked in river systems in Nova Scotia. Plots are labeled by river system and separated based on size. Coloured bars represent the fall (red) and spring (blue) stocking programs. Bars for stocking programs are positioned next to each other on x-axis (*i.e.*, not stacked or overlaid).

**
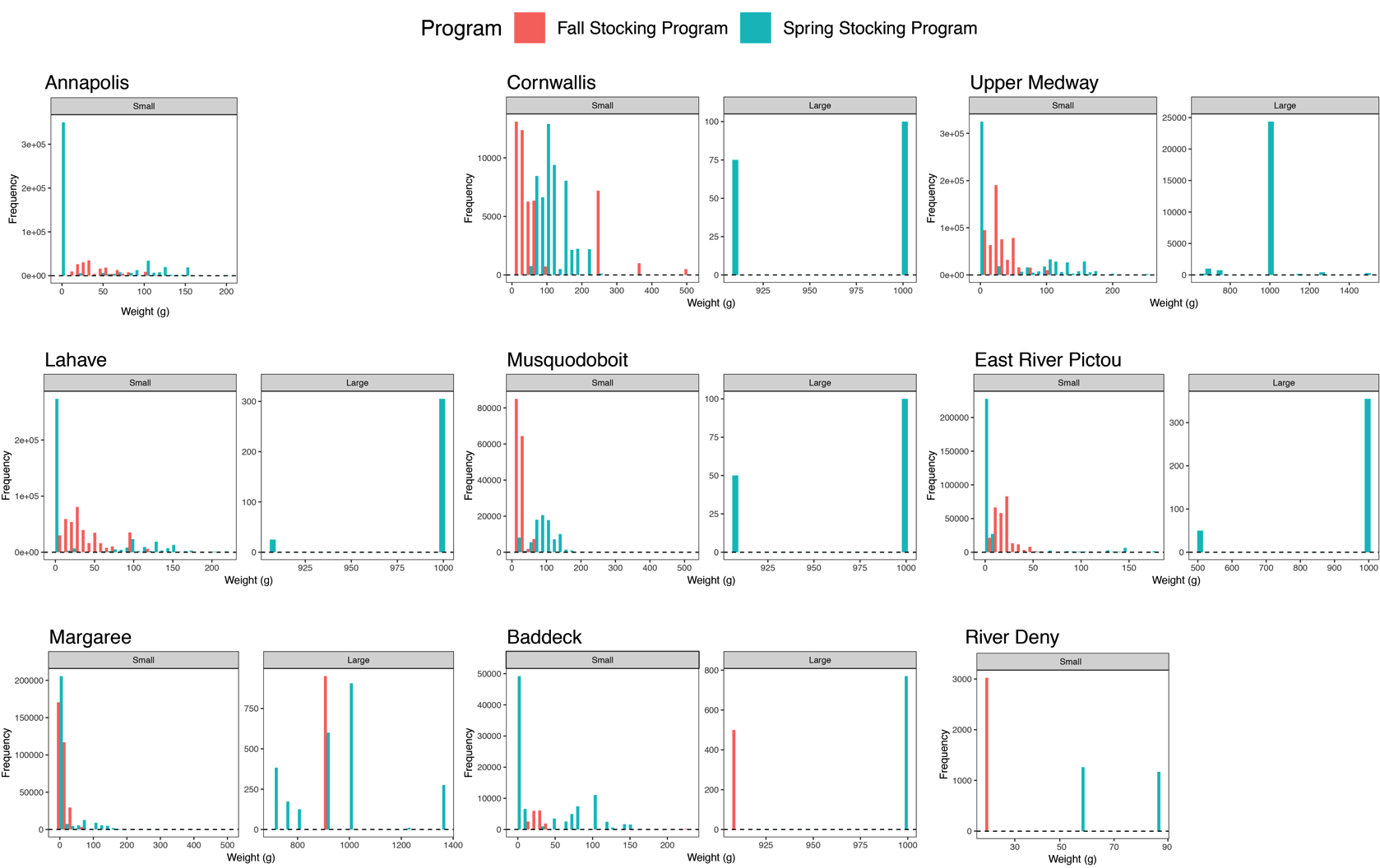
**

**Figure S2.** Number of brook trout (*Salvelinus fontinalis*) stocked in each year between 1976 and 2018 in each river system. Axis are all provided on the same scale for comparison. River system is indicated above each panel.


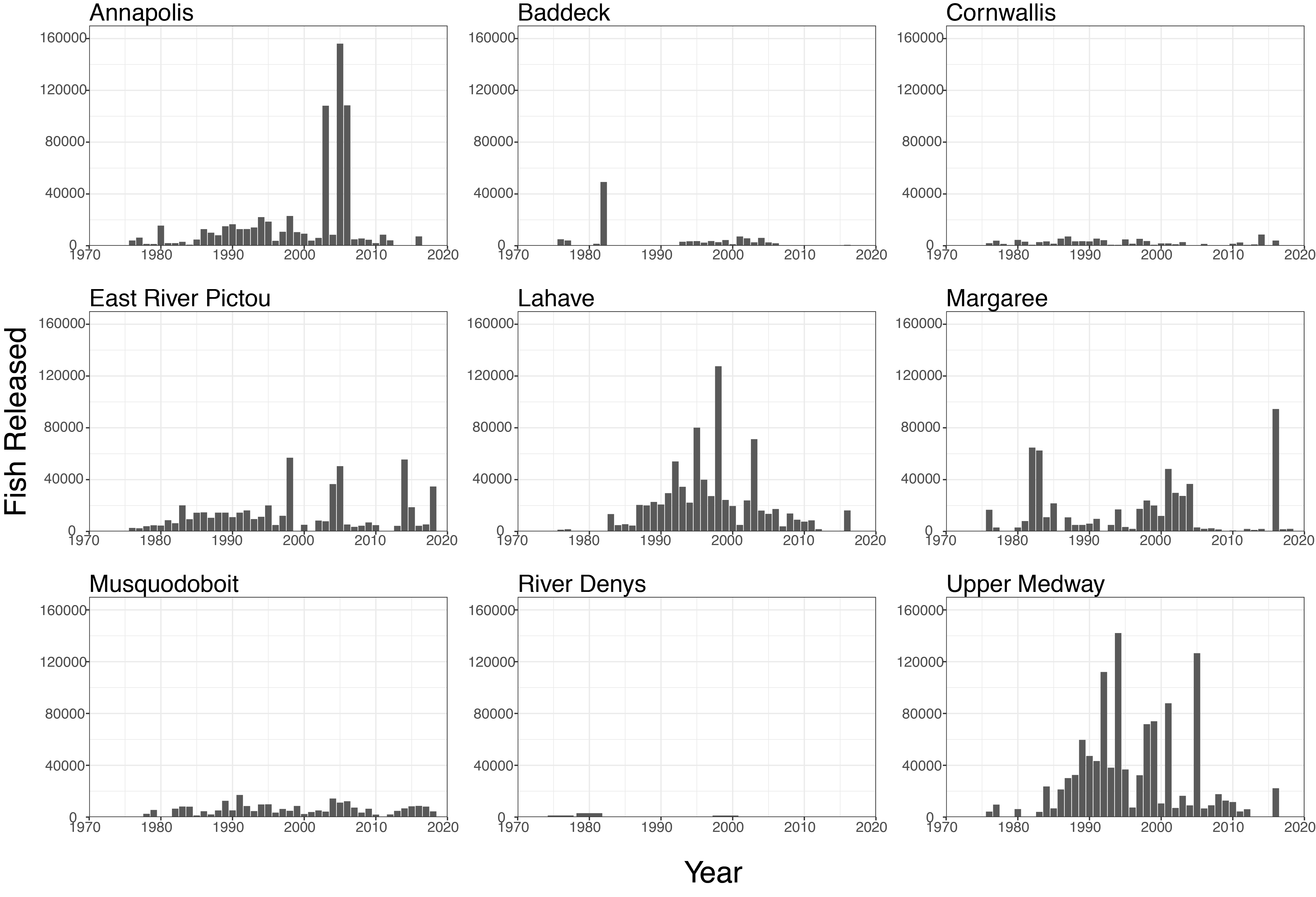


**Figure S3.** Genetic diversity estimates for wild brook trout (*Salvelinus fontinalis*) populations in Nova Scotia based on 100 microsatellites. **(A)** Estimates for inbreeding coefficient (*F*_IS_), **(B)** heterozygosity estimates (observed [*H*_o_; circle] and expected [*H*_e_; triangle]), and **(C)** effective population size (*N*_e_) are provided with 95% confidence intervals. *N*_e_ was calculated using an allele frequency cut-off (*P*-crit) of 0.01 and the corresponding confidence intervals were adjusted using the jackknife method. A maximum value of *N*_e_ was set at 4000 for plotting (but see Table 1 for all values) but higher values are indicated by arrows with infinite values indicated by ∞. All points and bars are coloured by river system and river systems are organized by longitude.

**
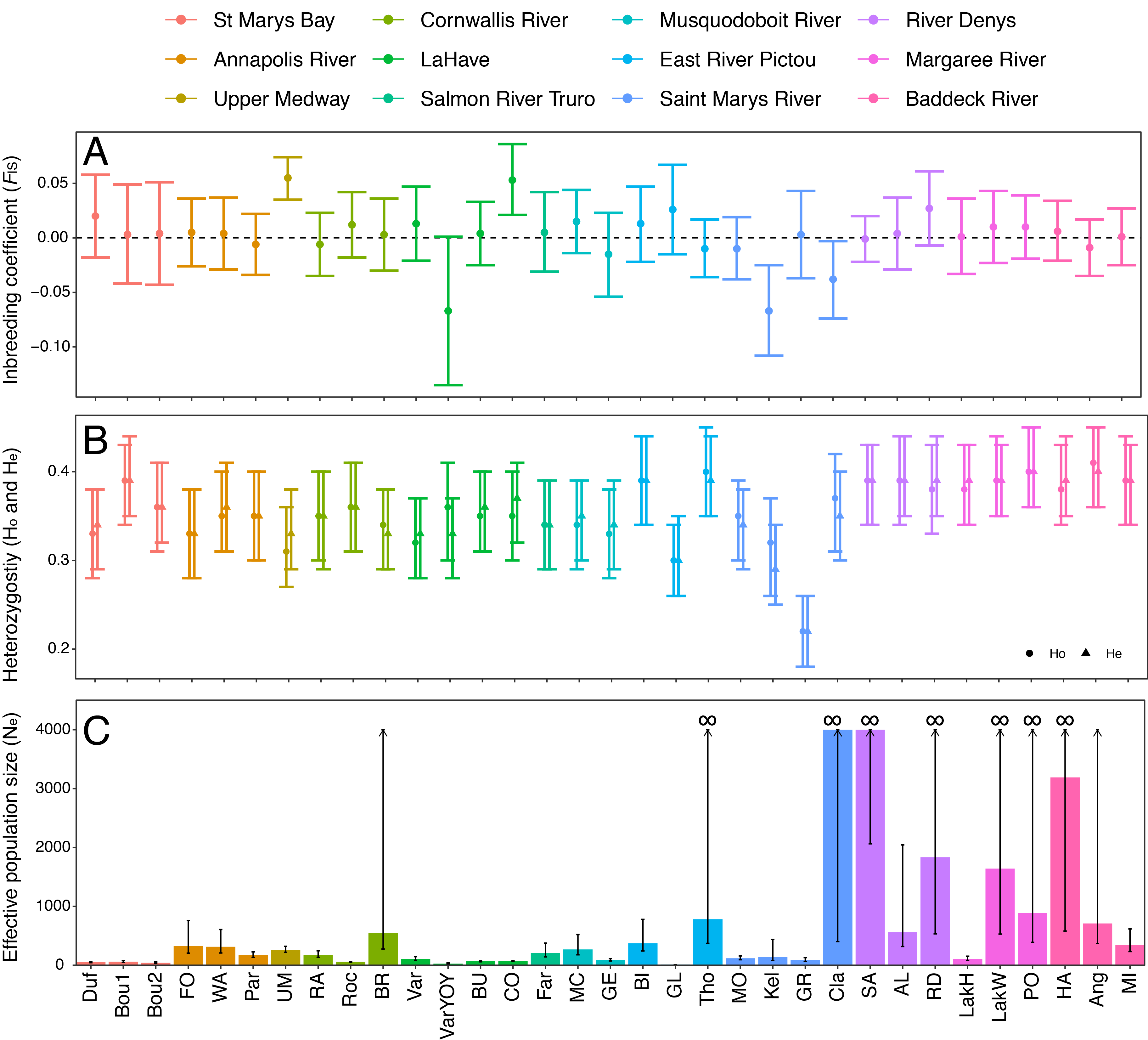
**

**Figure S4.** Relationship between genetic divergence (Slatkin’s linearized *F*_ST_) and distance (hydrologic distance) for wild brook trout (*Salvelinus fontinalis*) sites in Nova Scotia. Blue line indicates line of best fit. Relationship supports significant isolation-by-distance (p=0.001; r=0.23).
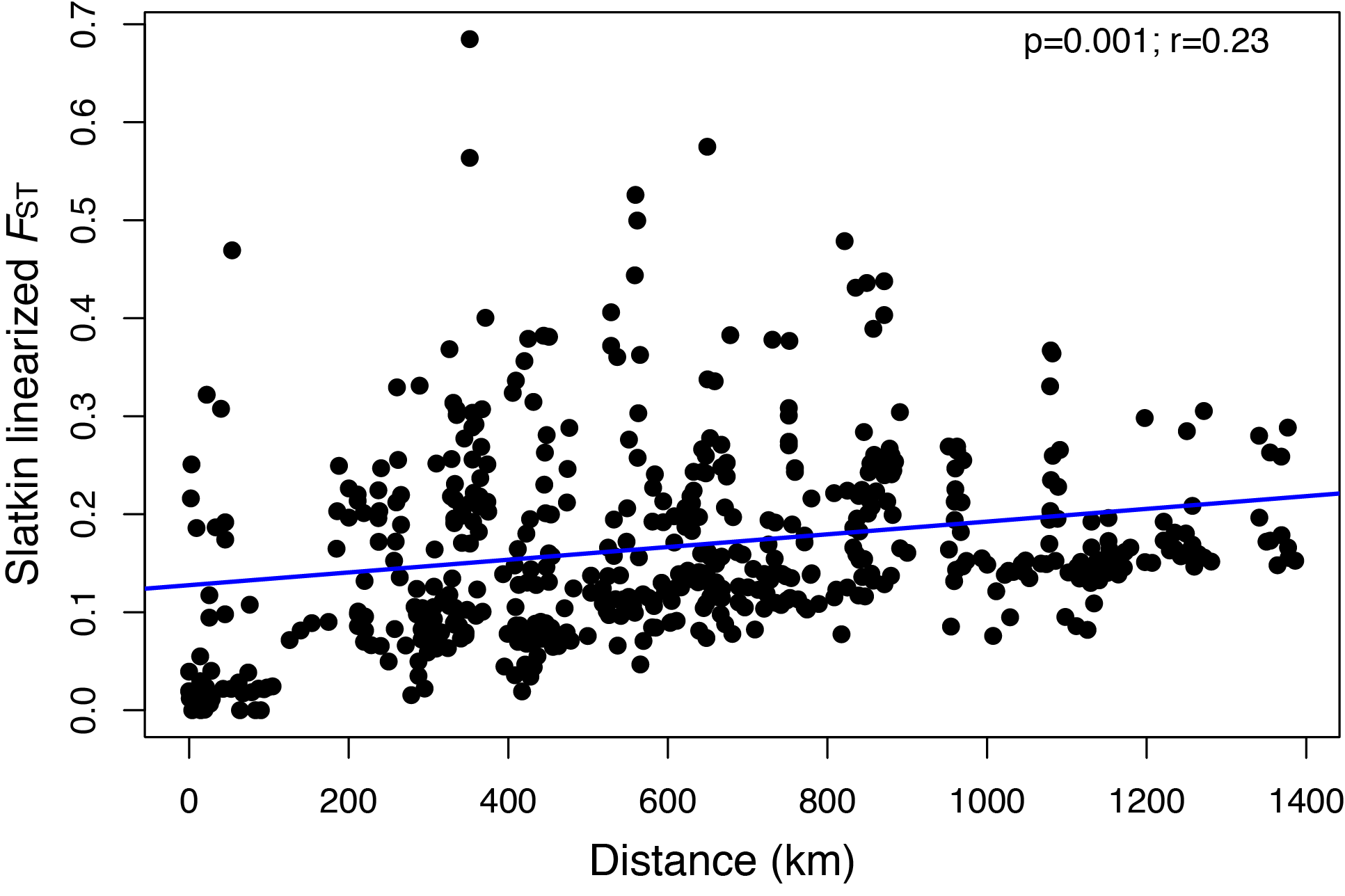


**Figure S5.** Principal coordinate analysis (PCoA) of brook trout (*Salvelinus fontinalis*) individuals from St. Mary’s Bay (wild) and Fraser’s Mill (hatchery).

**
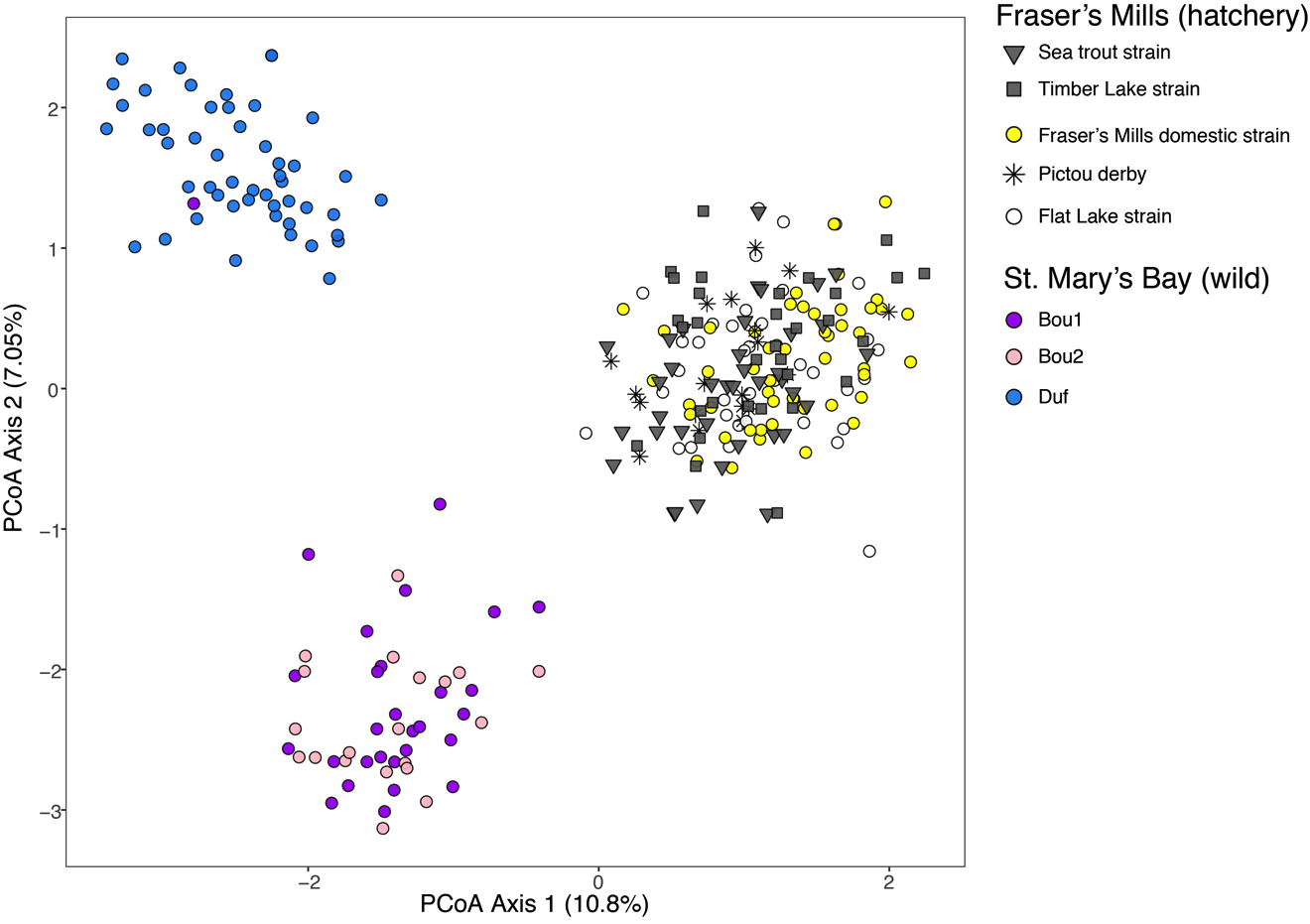
**

**Figure S6.** Output results from STRUCTURE HARVESTER v0.6.94 (Earl & vonHoldt, 2012) for STRUCTURE runs for brook trout (*Salvelinus fontinalis*) across Nova Scotia (K=1-40)*.* Mean LnPr(*X*|*K*) estimates across iterations and values of K to assess best K, where the arrow indicates the plateau at K=25.


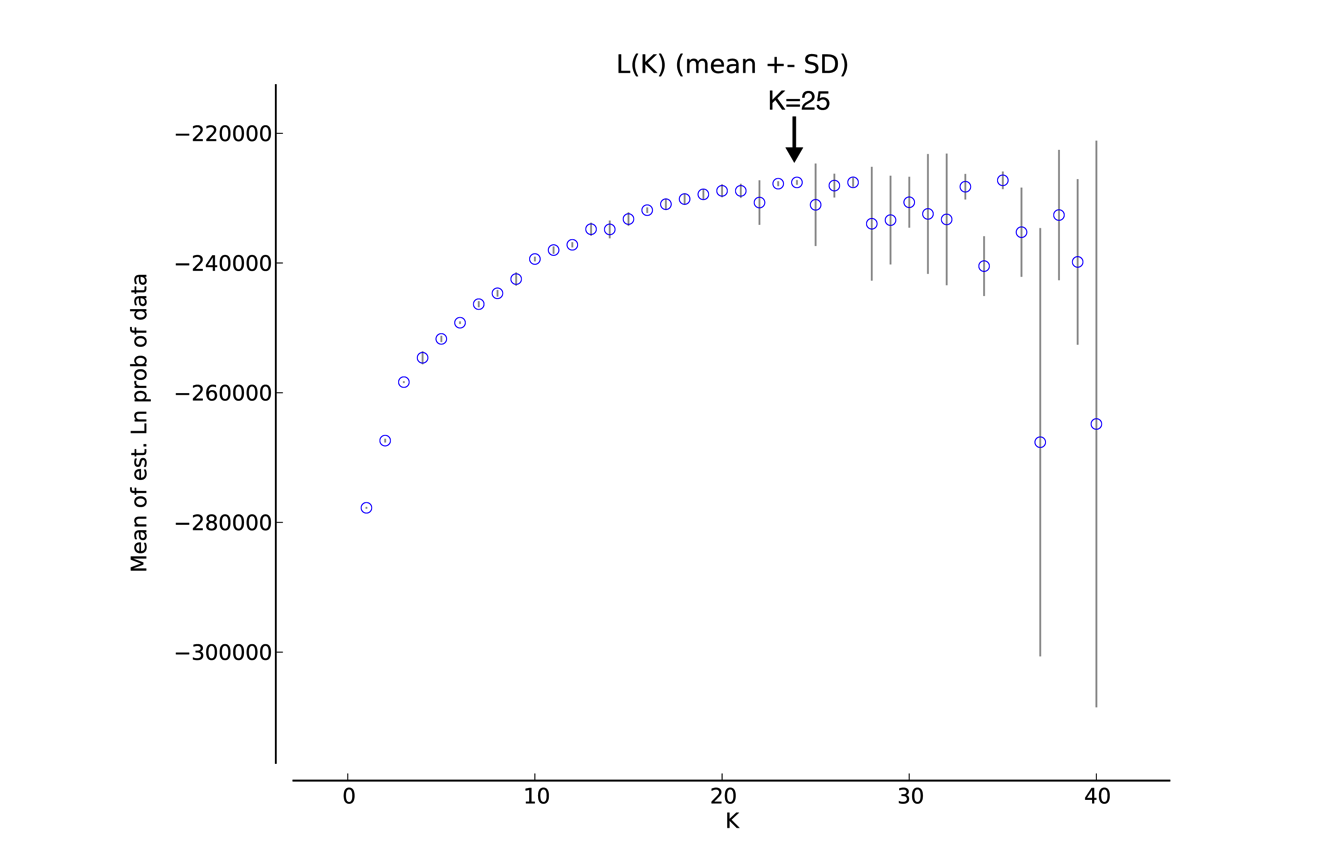


**Figure S7.** Contribution of 19 bioclimatic variables on principal component axes 1 to 3 with variables coloured by their relative contribution to the axes (see Figure S8), where darker orange and longer arrows indicates stronger contribution. Figures were generated using the R package *factoextra*.

**
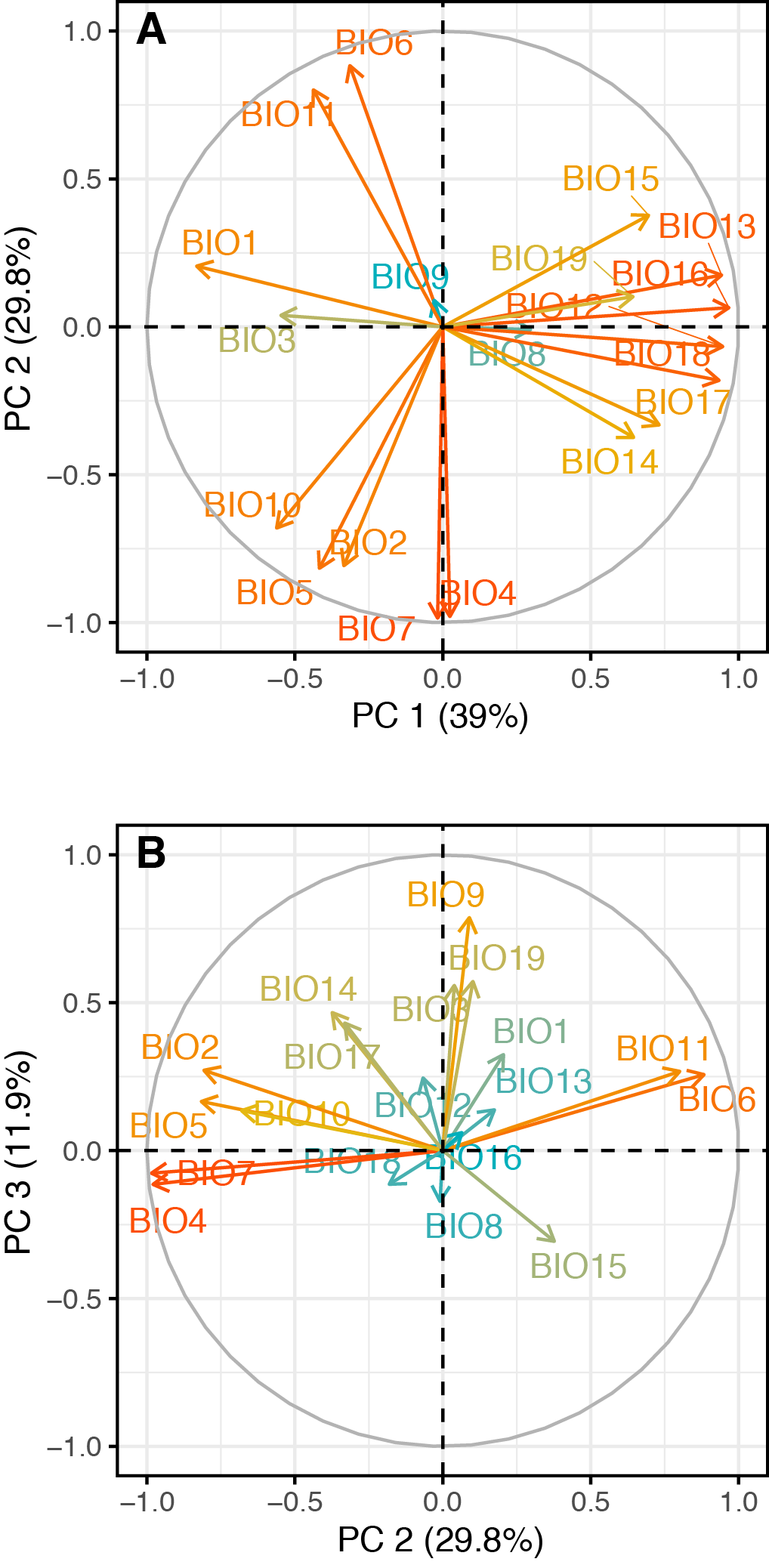
**

**Figure S8.** Loadings of 19 bioclimatic variables on principal component axes 1 to 3 with variables coloured by their absolute loading value on that respective axis.


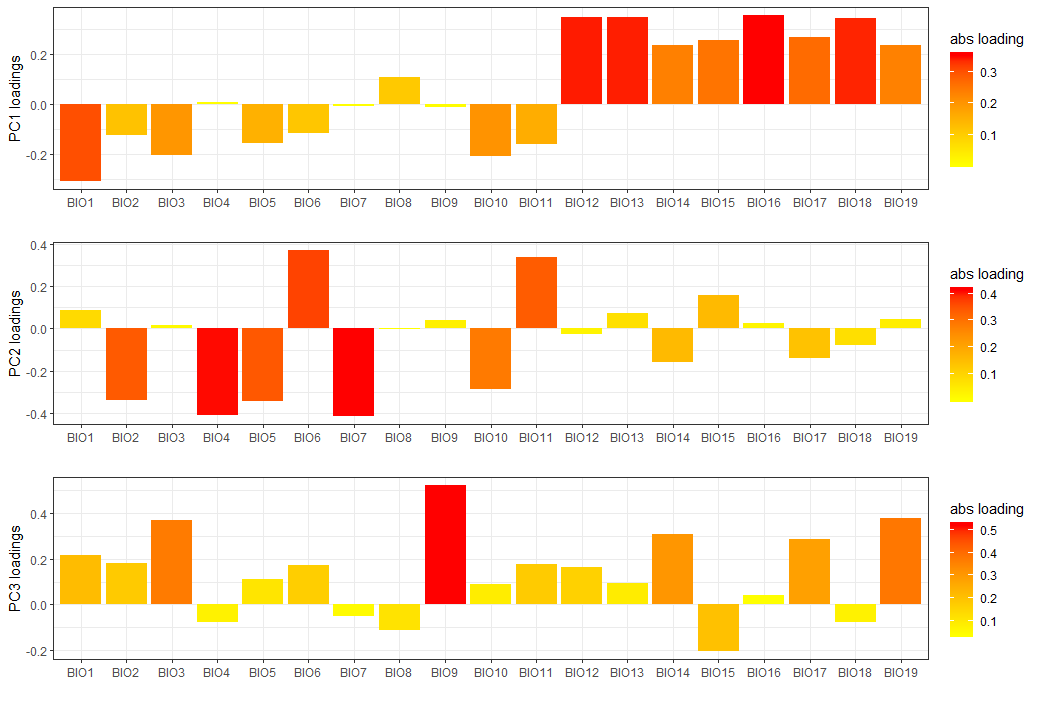


**Figure S9.** Contribution of 8 stocking variables on principal component axes 1 and 2 with variables coloured by their relative contribution to the axes (see Figure S10), where darker orange and longer arrows indicates stronger contribution. Figure was generated using the R package *factoextra*.

**
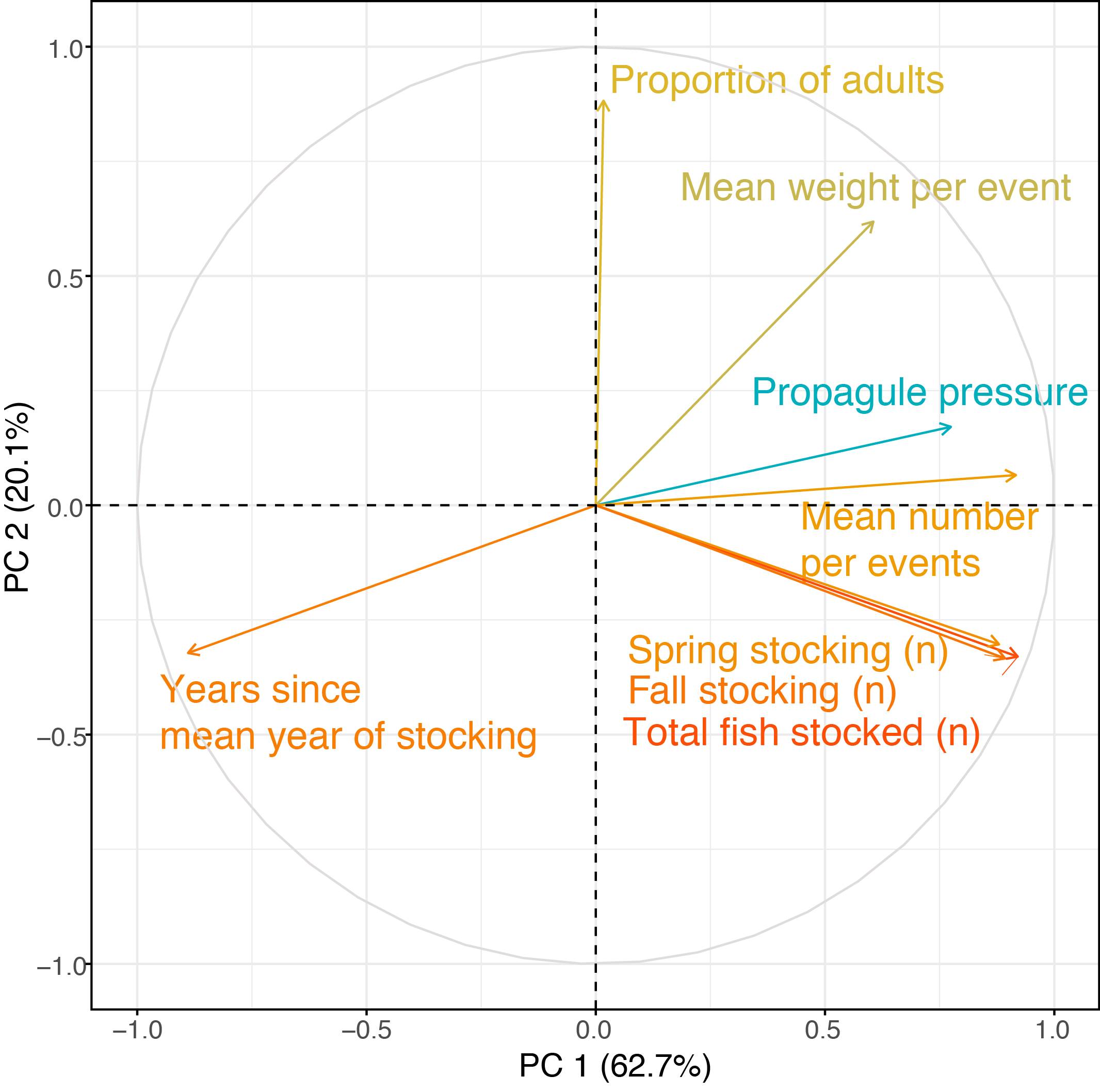
**

**Figure S10.** Loadings of 8 stocking variables on principal component axes 1 to 2 with variables coloured by their absolute loading value on that respective axis. Variables were abbreviated for the figure and correspond to total fish stocked in fall (Fall), mean number of fish stocked per event (MeanN), mean weight of fish stocked per event (MeanWt), total proportion of adults stocked (PropAdult), propagule pressure (PropPress), total fish stocked in spring (Spring), total fish stocked (TotalN), and years since mean year of stocking (YearSince).


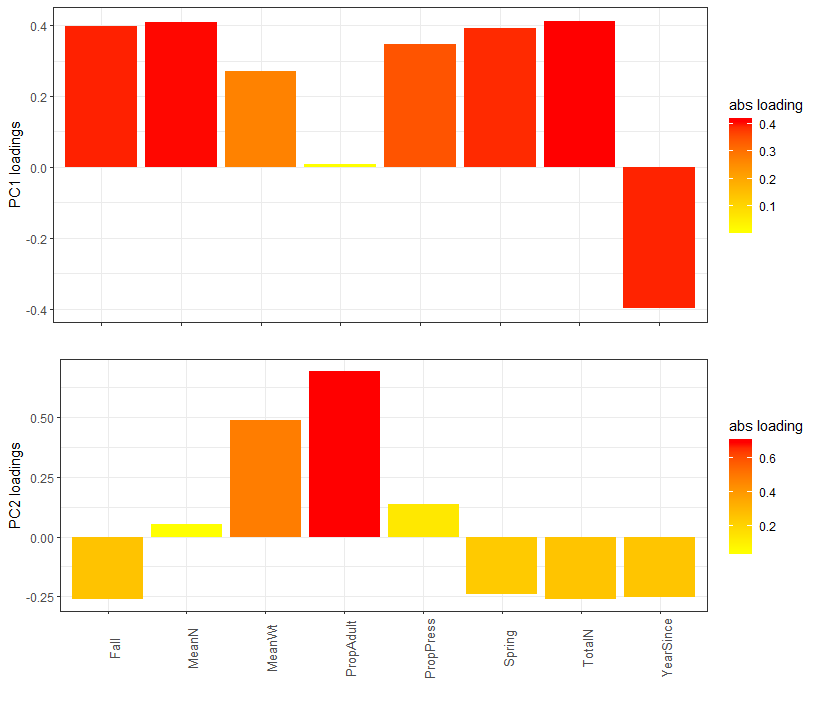


**Figure S11.** Relationship between significant variables identified by redundancy analysis and genetic population structure along principle coordinate (PCo) axis 2 for wild brook trout (*Salvelinus fontinalis*) populations in Nova Scotia. Panel **(A)** shows the relationship between genetic structure and proportion of adults stocked when all sites are included. Whereas panels **(B)** shows the same relationship when the Green’s Brook (GR) site is removed (see main text). Panels **(C,D)** show the relationship between genetic structure and bioclimatic variables including **(C)** BIO4 and **(D)** BIO7 where GR is excluded for both panels.

**
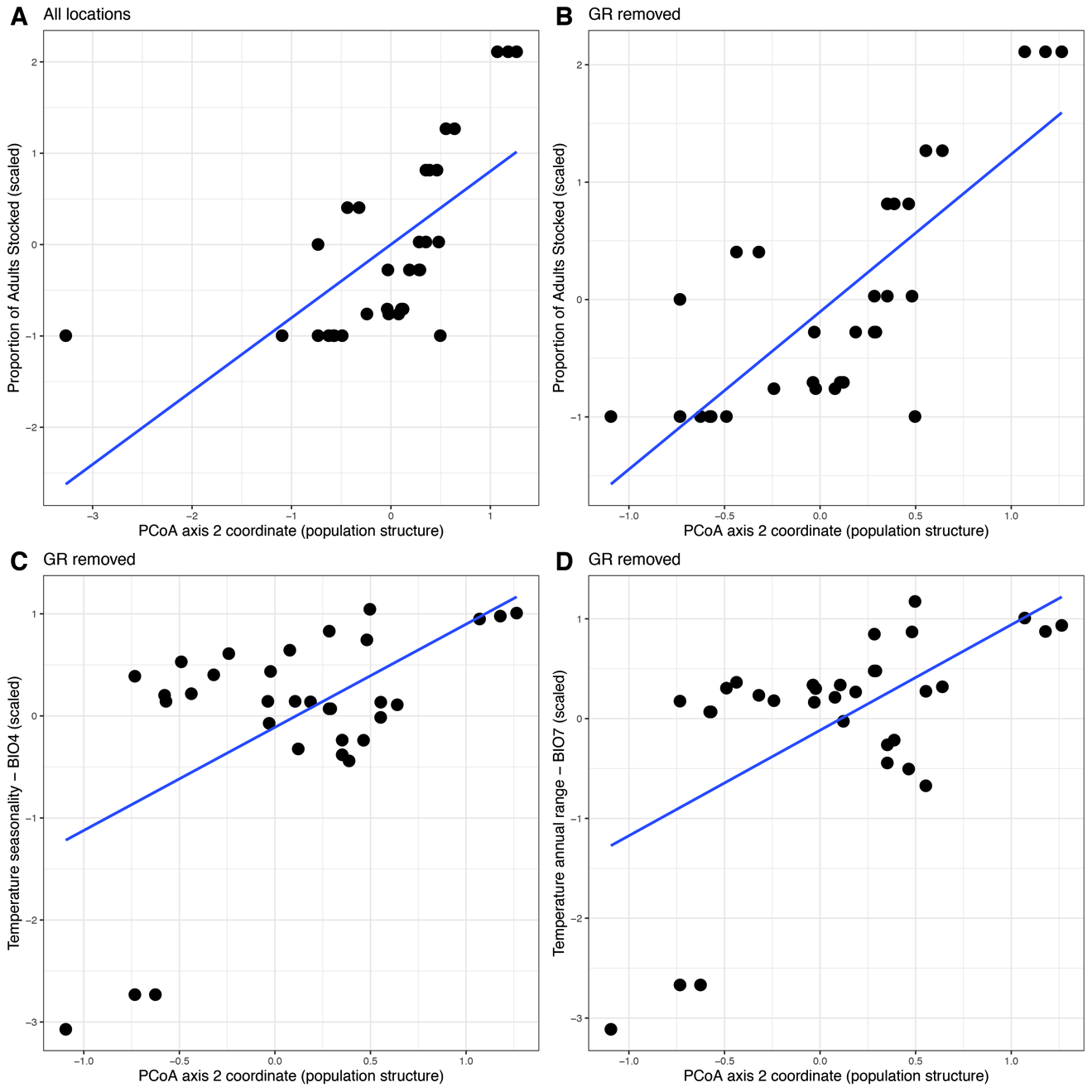
**
